# Prognostic value of the relative neutrophil–monocyte-to-lymphocyte–albumin ratio in chronic lower respiratory diseases: a multicenter retrospective analysis

**DOI:** 10.3389/fphys.2025.1708302

**Published:** 2025-12-18

**Authors:** Xu Chen, Yi Zhang, Xueyuan Wang, Liping Ye, Kaijia Shi, Xinghan Tian

**Affiliations:** 1 Department of Intensive Care Unit, Yantai Yuhuangding Hospital, Yantai, China; 2 Lishui Central Hospital, The Fifth Affiliated Hospital of Wenzhou Medical University, Lishui Hospital of Zhejiang University, Lishui, China

**Keywords:** NMLAR, chronic lower respiratory diseases, NHANES, machine learning, prognostic biomarker, inflammation

## Abstract

**Background:**

Chronic lower respiratory diseases (CLRDs) remain major causes of global mortality. Because conventional inflammatory markers have limited prognostic utility, we developed and validated the relative neutrophil–monocyte–lymphocyte–albumin ratio (NMLAR), defined as (Neutrophil% × Monocyte% × 100)/(Lymphocyte% × Albumin [g/dL]), as a novel biomarker to predict CLRD-specific mortality.

**Methods:**

Immune infiltration of CLRDs was analyzed based on GEO datasets. We then analyzed 9,236 adults with CLRD from NHANES 1999–2014, excluding individuals with missing core variables. Machine learning algorithms (Boruta, SVM-RFE, XGBoost) were applied to identify key predictors. Cox proportional hazards models and restricted cubic spline (RCS) functions were used to evaluate the association between NMLAR and mortality outcomes, and stratified analyses were conducted across clinically relevant subgroups. Model performance was assessed by Harrell’s C-index, calibration plots, and decision-curve analysis (DCA). Findings were externally validated in NHANES 2015–2018 (n = 2,107), the MIMIC-IV v3.1 ICU cohort (n = 2,120), and a real-world Zhejiang Provincial ICU cohort (n = 161).

**Results:**

Immune profiling showed increased neutrophils/monocytes and reduced lymphocytes in CLRD and acute states. Higher baseline NMLAR was consistently associated with increased risks of both all-cause and CLRD-specific mortality and demonstrated superior predictive performance compared with conventional inflammatory markers. In NHANES, fully adjusted models indicated an approximately linear dose–response, with each 1-unit increment in NMLAR corresponding to a ∼7% higher risk of all-cause mortality and an ∼8% higher risk of CLRD-specific mortality. In the MIMIC cohort, NMLAR remained independently associated with 14–365-day mortality even after adjustment for critical care–specific covariates (SOFA score, CRRT, invasive mechanical ventilation, vasopressor use), with a threshold effect identified at 12.10. In the Zhejiang ICU cohort, NMLAR independently predicted 30-day mortality (HR per unit increase ≈1.09), with a threshold at 13.32. Notably, models derived from NHANES demonstrated moderate discriminatory ability, satisfactory calibration, and clinical net benefit when externally validated in both ICU cohorts, underscoring the robustness and generalizability of NMLAR as a prognostic biomarker across diverse clinical settings.

**Conclusion:**

NMLAR is a simple, robust, and clinically applicable biomarker for mortality risk in CLRD, demonstrating consistent prognostic value across population-based, critical care, and real-world cohorts.

## Introduction

Chronic lower respiratory diseases (CLRDs), including COPD, emphysema, chronic bronchitis, and asthma (Lee et al., 2021), remain a leading cause of morbidity and mortality worldwide (Prevalence and attributable health burden of chronic respiratory diseases, 2020). Broadly defined, COPD encompasses emphysema, chronic bronchitis, and chronic obstructive asthma, with COPD and asthma representing the most prevalent chronic airway diseases (Cheng et al., 2021; Li et al., 2024). Despite being classified as distinct clinical entities, these conditions are intrinsically linked by their common pathological basis—inflammation of the airways. It is estimated that approximately 30% of patients with COPD and 26% of those with asthma exhibit features of ACO (Dey et al., 2022). Accordingly, the present study focuses primarily on these two conditions. In 2020, CLRD accounted for one of the five leading causes of mortality in the United States (Baral et al., 2024). Globally, over 200 million individuals are estimated to have COPD, with more than 3 million deaths annually attributed to it (GBD, 2019 Ch ronic Respiratory Diseases Collaborators, 2023). Despite therapeutic advances, effective risk stratification remains challenging due to the heterogeneity of CLRD and the interplay of inflammatory, environmental, and genetic factors.

Systemic immune–inflammation plays a crucial role in the development and progression of chronic lung diseases (CLRD) (Bhatt et al., 2024). In recent years, numerous inflammation-related indices derived from routine complete blood counts—such as the neutrophil–lymphocyte ratio (NLR), platelet–lymphocyte ratio (PLR), monocyte–lymphocyte ratio (MLR), and several composite hematologic inflammation scores—have demonstrated significant prognostic value across a broad spectrum of chronic diseases (Cai et al., 2024). These biomarkers are closely associated with coronary disease severity, major adverse cardiovascular events (MACE), and in-hospital mortality, and they also reflect disease activity, exacerbation risk, and survival outcomes in COPD and other chronic respiratory disorders, thereby supporting their utility in clinical risk stratification (Tudurachi et al., 2023; Zhang et al., 2024). Similar predictive performance has been reported in oncology, where such inflammation-based indices can independently predict overall survival in colorectal cancer and, in some studies, even outperform conventional TNM staging systems (Yang et al., 2023). Collectively, current evidence underscores the strengths of blood cell–derived inflammatory markers—rapid availability, low cost, and high reproducibility—as practical tools for evaluating systemic inflammation. However, most widely used indices, such as NLR, PLR, and MLR, are constructed from absolute leukocyte counts. Although useful, they mainly reflect quantitative changes and tend to overlook subtle but meaningful alterations in relative immune-cell proportions. In CLRD, where inflammatory phenotypes are heterogeneous and immune regulation is highly dynamic, proportional shifts in neutrophils, monocytes, and lymphocytes may provide a more sensitive indication of immune dysregulation than absolute counts alone. This limitation may partly explain the modest performance of traditional indices in disease-specific risk prediction. To address this gap, we developed the neutrophil–monocyte to lymphocyte–albumin ratio (NMLAR), a percentage-based index designed to better capture relative changes in leukocyte composition. NMLAR is calculated as (Neutrophil% × Monocyte% × 100)/(Lymphocyte% × Albumin [g/dL]).

NHANES, with its nationally representative sampling design, standardized measurements, and linkage to the National Death Index enabling precise identification of CLRD-specific mortality, provides an ideal platform for evaluating inflammation–nutrition biomarkers in the general population. In parallel, machine learning (ML) methods have gained traction in large-scale epidemiological research. Compared with traditional regression models, ML algorithms are well-suited to capture nonlinear relationships and high-dimensional interactions, thereby improving predictive accuracy and generalizability (Liao et al., 2025). However, their application in predicting CLRD-specific mortality—particularly within nationally representative datasets such as NHANES—remains limited. To fill this gap, we leveraged NHANES data from 1999 to 2014 to assess the prognostic utility of NMLAR for both CLRD-specific and all-cause mortality. Three commonly used ML algorithms—Boruta, SVM-RFE, and XGBoost—were applied to identify key prognostic variables (Hu et al., 2024). We then evaluated the association and dose–response relationship between NMLAR and mortality outcomes using Cox proportional hazards models and restricted cubic splines. Findings were externally validated in three independent cohorts: NHANES 2015–2018, the MIMIC-IV ICU cohort, and the Zhejiang Provincial ICU cohort. Collectively, this study provides novel evidence supporting NMLAR as a clinically meaningful biomarker for risk stratification in CLRD populations, applicable to both general and critically ill patients.

## Methods

### Study design and population

We first conducted immune-infiltration analysis to evaluate and compare immune cell proportions in chronic lower respiratory disease (CLRD), incorporating four GEO datasets: GSE16972 (alveolar macrophages from COPD patients), GSE27876 (peripheral blood cells from asthma patients), GSE60399 (PBMC samples from stable COPD and AECOPD patients collected on hospital days 1, 3, and 10), and GSE184693 (rat COPD model induced by cigarette smoke plus intratracheal LPS, with NaHS or PPG interventions). Raw data were processed into gene expression matrices; Affymetrix datasets were normalized and annotated using RMA, while author-provided matrices were used for PBMC samples. CIBERSORT (LM22 signature, 22 immune-cell subsets, 1,000 permutations) was then applied, and only samples with deconvolution P < 0.05 were retained for downstream analyses and visualization.

This retrospective cohort study integrated three complementary datasets. The primary development cohort was derived from NHANES 1999–2014. Participants were included if they self-reported at least one CLRD, including COPD, emphysema, chronic bronchitis, or asthma, or reported persistent cough with sputum production for at least 3 months per year. Exclusion criteria were age <18 years or missing mortality status and key laboratory variables. Missing covariate data were imputed using the K-nearest neighbor (KNN) algorithm. A total of 9,236 adults were included for model development and internal validation. Mortality outcomes were determined by linkage to the National Death Index (NDI) with follow-up through December 31, 2019 (Gao et al., 2025). The primary outcomes were all-cause mortality and CLRD-specific mortality, identified using ICD-10 codes J40–J47 (including asthma, chronic bronchitis, emphysema, and COPD). An independent temporal validation cohort was established from NHANES 2015–2018 using the same criteria.

To assess generalizability in patient cohorts, we used the MIMIC-IV v3.1 adult ICU database. Eligible patients were those with a primary diagnosis of CLRD, including COPD, asthma, and emphysema, as well as acute exacerbations identified through an *a priori* ICD-9/10 code set (e.g., J449, J441, J440). Among 8,716 candidate ICU admissions, 15 patients with hematologic malignancies (e.g., ICD-9 20510, 20502, 20401) and 6,581 with missing NMLAR data were excluded, leaving 2,120 patients. Baseline laboratory measurements were defined as the values closest to ICU admission within ±24 h. NMLAR was two-sided trimmed at the 1st and 99th percentiles to mitigate outlier effects. Smoking status was classified as current, former, or never smoker based on ICD-9/10 inpatient codes. The primary endpoints were all-cause mortality at 7, 14, 30, 90, and 365 days after ICU admission.

**FIGURE 1:**
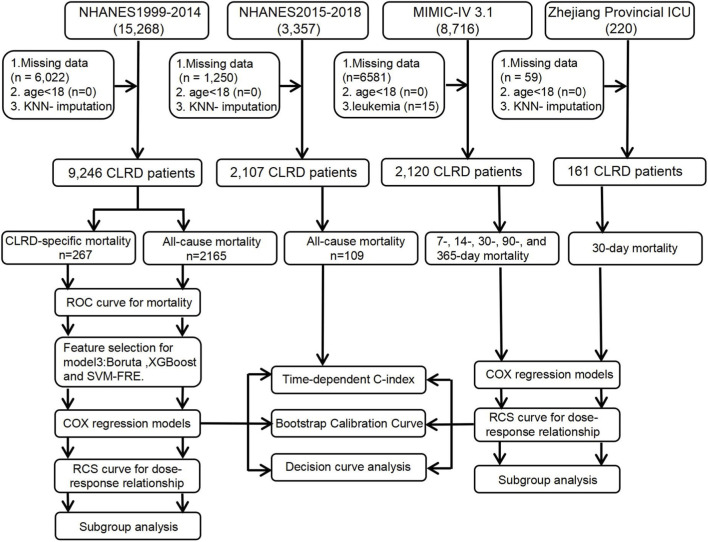
Flowchart of the study.

Finally, we constructed an independent real-world validation cohort from the Zhejiang Provincial ICU electronic health record (EHR) system (Jin et al., 2023). Using the same CLRD diagnostic framework, 220 hospitalized patients were screened. After excluding 59 cases with missing core exposure or covariates, 161 patients were included. Baseline laboratory variables were defined as the measurements nearest to ICU admission within the first 24 h, aligned with the NHANES and MIMIC definitions. The primary outcome was 30-day all-cause mortality (Figure 1).

### Covariate definitions

A comprehensive set of covariates associated with CLRD and mortality risk were included. Age was analyzed as a continuous variable, while sex (male/female) and race/ethnicity (Mexican American, other Hispanic, non-Hispanic White, non-Hispanic Black, other/multiracial) were categorical. Education was classified as < high school, high school graduate, or > high school. Socioeconomic status was measured by poverty income ratio (PIR; low <1.5, middle 1.5–4.0, high >4.0). Body mass index (BMI) was calculated from height and weight and categorized as underweight (<18.5 kg/m^2^), normal (18.5–24.9), overweight (25.0–29.9), and obese (≥30.0). Smoking status was defined as current, former, or never, and alcohol intake was harmonized into monthly drinking frequency (non-drinker, 1–5, 5–10, ≥10 times/month, or uncertain). Comorbidities (yes/no) included hypertension, heart failure, coronary heart disease, stroke, cancer, liver disease, chronic kidney disease, arthritis, gout, asthma, chronic bronchitis, emphysema, and COPD. Diabetes was defined by self-report, medication use, or laboratory criteria (HbA1c ≥6.5%, fasting glucose ≥126 mg/dL, or OGTT ≥200 mg/dL); prediabetes was defined as HbA1c 5.7%–6.4%, fasting glucose 100–125 mg/dL, or OGTT 140–199 mg/dL; others were normoglycemic. Hyperlipidemia was based on self-report, medication use, or labs (total cholesterol ≥240 mg/dL, LDL-C ≥160 mg/dL, HDL-C <40 mg/dL, or triglycerides ≥200 mg/dL). Laboratory covariates included hemoglobin (HGB), white blood cell count (WBC), platelet count (PLT), albumin (ALB), creatinine (Cr), blood urea nitrogen (BUN), total bilirubin (TBIL), sodium (Na), potassium (K), calcium (Ca), alanine aminotransferase (ALT), aspartate aminotransferase (AST), lactate dehydrogenase (LDH), serum osmolality (OSM), and C-reactive protein (CRP). Inflammation-related indices were also derived and analyzed.

### Definition of inflammatory markers

NMLAR = (Neutrophil% × Monocyte% × 100)/(Lymphocyte% × Albumin [g/dL]).

NAPR = Neutrophil%/Albumin [g/dL] (Su et al., 2025).

SIRI = (Neutrophil Count [10^3^/μL] × Monocyte Count [10^3^/μL])/Lymphocyte Count [10^3^/μL] (Zhang and Cheng, 2024).

PLR = Platelet Count [10^3^/μL]/Lymphocyte Count [10^3^/μL] (Tan et al., 2024).

NLR = Neutrophil Count [10^3^/μL]/Lymphocyte Count [10^3^/μL] (Fu et al., 2025).

MLR = Monocyte Count [10^3^/μL]/Lymphocyte Count [10^3^/μL] (Fu et al., 2025).

### Statistical analysis

All statistical analyses were performed using R software (version 4.4.0). The proportion of missing values for all included variables was <30% and imputed using the KNN algorithm. Baseline characteristics were summarized according to NMLAR quartiles: continuous variables were expressed as medians with interquartile ranges [Q1, Q3] and compared using one-way ANOVA or Kruskal–Wallis H tests, while categorical variables were presented as frequencies (%) and compared using chi-square tests. The discriminatory and predictive ability of NMLAR and other inflammatory indices for both CLRD-specific and all-cause mortality were assessed using ROC curves, with DeLong’s test applied for AUC comparisons. To identify important predictors associated with mortality outcomes, three machine-learning algorithms—SVM-RFE, XGBoost, and Boruta—were applied separately (Fu et al., 2024), and the top 10 predictors from each algorithm were combined to form a unified feature set (Liao et al., 2025). Cox proportional hazards models were then constructed with NMLAR included as both a continuous and categorical variable (quartiles): Model 1 was unadjusted, Model 2 adjusted for age and sex, and Model 3 further adjusted for the unified feature set. Restricted cubic splines (RCS) were fitted to explore potential nonlinear dose–response relationships between NMLAR and mortality risk. The optimal cutoff value of NMLAR was determined using the Youden index to stratify participants into high- and low-risk groups. Subgroup analyses and sensitivity analyses were conducted according to demographic and disease characteristics. Model performance was evaluated by discrimination (time-dependent C-index), calibration (calibration curves with 1,000 bootstrap resamples at 60 months), and clinical utility (decision curve analysis, DCA) (Liao et al., 2025; Wan et al., 2025). Finally, an internal temporal validation was conducted using the NHANES 2015–2018 cohort of participants with CLRD to further assess model robustness and generalizability.

We externally validated the prognostic value of NMLAR in two ICU cohorts with different analytic frameworks. In the MIMIC-IV v3.1 cohort, Cox proportional-hazards models were constructed in three stages: Model 1 included NMLAR alone; Model 2 additionally adjusted for demographic and laboratory covariates aligned with NHANES; and Model 3 further incorporated ICU-specific variables including SOFA score, continuous renal replacement therapy (CRRT), invasive mechanical ventilation, and vasopressor use. Mortality endpoints were assessed at 7, 14, 30, 90, and 365 days after ICU admission. In the Zhejiang Provincial ICU cohort, models were specified in accordance with the NHANES framework but tailored to the available sample size and follow-up horizon. Cox models included NMLAR alone (Model 1), adjustment for demographic and laboratory covariates (Model 2), and further adjustment for comorbidities (Model 3). The primary outcome was 30-day all-cause mortality. Model performance was evaluated using AUC, Harrell’s C-index, calibration curves, and DCA. RCS was fitted within the Cox framework to examine potential nonlinear associations between NMLAR and mortality, and subgroup analyses were performed to test the robustness of findings across clinically relevant strata. In the Zhejiang ICU EHR system, smoking status could not be ascertained; therefore, for model evaluation, we introduced an alternative Model 4 (assuming all patients were smokers) and compared it with Model 3 (assuming all patients were non-smokers).

## Results

### Assessment of immune cell infiltration

**FIGURE 2:**
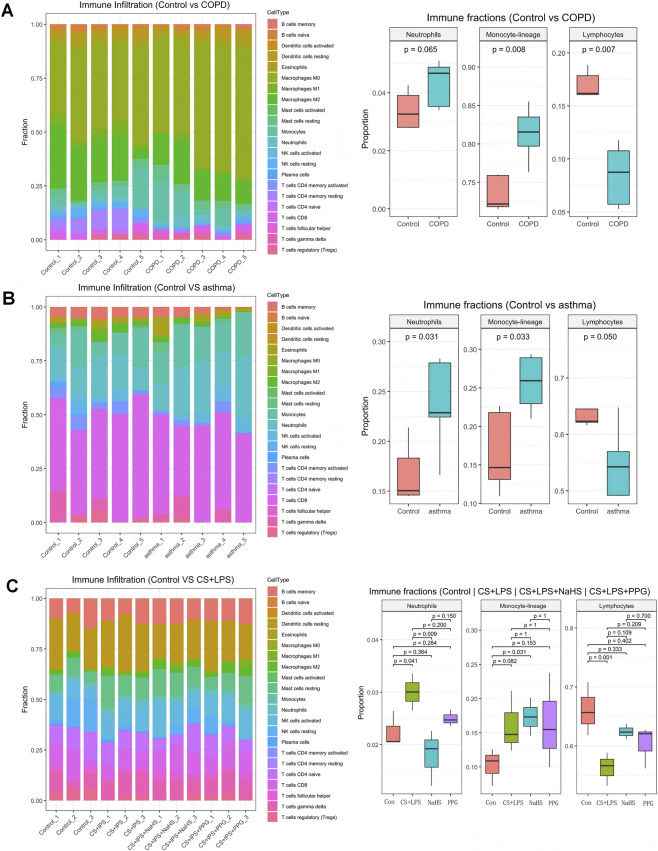
Immune cell infiltration in CLRD. CIBERSORT (LM22) analysis showed higher monocyte-lineage and neutrophil fractions with reduced lymphocytes in COPD **(A)** and asthma **(B)**. In the rat CS + LPS model **(C)**, innate immune fractions increased and lymphocytes decreased; NaHS partly reversed these changes, while PPG reinforced them.

Using CIBERSORT (LM22) deconvolution (Figure 2), we compared relative immune-cell fractions between CLRD cohorts and controls. In COPD (Figure 2A), monocyte-lineage fractions were significantly higher (p = 0.008), lymphocytes were lower (p = 0.007), and neutrophils showed a trend toward increase (p = 0.065). In asthma (Figure 2B), both neutrophil and monocyte-lineage fractions were elevated (p = 0.031 and 0.033), while lymphocytes were reduced with borderline significance (p = 0.050). In the rat CS + LPS model (Figure 2C), innate fractions (neutrophils, monocytes) increased and lymphocytes decreased; NaHS partially reversed these changes, whereas PPG reinforced them. Results in AECOPD (Supplementary Figure S1) showed the same directionality. Collectively, these findings indicate that immune-cell fraction profiles sensitively reflect CLRD-associated disease states.

### Baseline characteristics of the study population

A total of 9,236 participants with CLRD were included in this study. Participants were categorized into quartiles based on NMLAR levels, and their baseline characteristics are presented in Table 1. As NMLAR increased, the median age rose from 43 years (IQR: 30–55) in Q1 to 56 years (IQR: 41–71) in Q4 (p < 0.001). The proportion of females declined across quartiles, from 63% in Q1 to 48% in Q4 (p < 0.001). Regarding race/ethnicity, the proportion of non-Hispanic White participants increased with higher NMLAR, while that of Mexican Americans and non-Hispanic Black participants decreased (p < 0.001). Educational attainment differed significantly among groups (p = 0.003), with fewer individuals in Q4 having education beyond high school. Similarly, the proportion of low PIR was higher in Q4 (p = 0.015). A higher NMLAR was associated with increased prevalence of comorbidities including CKD, arthritis, COPD, hypertension, HF, CHD, and cancer (all p < 0.001). The prevalence of diabetes rose from 12% in Q1 to 19% in Q4, whereas the prevalence of asthma declined with increasing NMLAR (p < 0.001). In terms of health behaviors, the proportions of current and never smokers decreased across NMLAR quartiles, while the proportion of former smokers increased (p < 0.001). Participants reporting heavy alcohol consumption (≥10 times/month) were more common in Q4 (22%) compared to Q1 (17%) (p = 0.001). For laboratory findings, levels of LDH, WBC, Cr, BUN, and CRP were significantly elevated in higher NMLAR quartiles, while Alb levels showed a decreasing trend (all p < 0.001). Follow-up duration was shorter in participants with higher NMLAR (p < 0.001). Composite inflammatory indices including NLR, MLR, SIRI, lgSII, lgPLR, and NAPR increased consistently across quartiles (all p < 0.001). Notably, CLRD-specific mortality rose from 1.0% in Q1 to 5.4% in Q4 (p < 0.001). In the NHANES 2015–2018 cohort (Supplementary Table S1), similar baseline characteristics were observed, with deceased participants exhibiting significantly higher NMLAR levels (P < 0.001).

**TABLE 1:** Baseline characteristics stratified by NMLAR.

NMLAR (N = 9236)^a^	Quartile 1 (≤2.64)	Quartile 2 (2.64–3.65)	Quartile 3 (3.65–5.13)	Quartile 4 (≥5.13)	p.value^b^
Age	43 (30, 55)	45 (31, 57)	48 (35, 62)	56 (41, 71)	<0.001
Gender%					<0.001
Female	1,419 (63%)	1,294 (55%)	1,229 (54%)	1,095 (48%)	
Male	890 (37%)	1,015 (45%)	1,080 (46%)	1,214 (52%)	
Race%					<0.001
Mexican american	288 (5.2%)	304 (5.1%)	285 (4.2%)	234 (3.8%)	
Other hispanic	183 (5.2%)	176 (5.3%)	173 (4.9%)	141 (4.1%)	
Non-hispanic white	1,070 (71%)	1,371 (80%)	1,471 (83%)	1,570 (84%)	
Non-hispanic black	768 (18%)	458 (9.5%)	380 (7.9%)	364 (8.0%)	
Education%					0.003
<high school	692 (22%)	661 (20%)	628 (18%)	713 (23%)	
High school	491 (23%)	506 (23%)	555 (26%)	535 (25%)	
>high school	1,126 (54%)	1,142 (57%)	1,126 (56%)	1,061 (52%)	
PIR%					0.015
low (<1.5)	1,027 (34%)	928 (29%)	905 (28%)	967 (31%)	
middle (1.5–4.0)	808 (39%)	872 (40%)	867 (40%)	900 (41%)	
high (>4.0)	474 (28%)	509 (31%)	537 (32%)	442 (28%)	
BMI%					0.056
underweight (<18.5)	51 (2.7%)	49 (2.0%)	31 (1.3%)	47 (2.1%)	
normal (18.5–24.9)	656 (29%)	588 (26%)	599 (28%)	637 (29%)	
overweight (25.0–29.9)	674 (30%)	710 (32%)	723 (29%)	744 (32%)	
obese (>30.0)	928 (38%)	962 (40%)	956 (41%)	881 (37%)	
Smoker%					<0.001
Never smoker	1,101 (43%)	1,108 (47%)	1,050 (44%)	884 (37%)	
Former smoker	492 (22%)	538 (23%)	625 (26%)	843 (34%)	
Current smoker	716 (35%)	663 (30%)	634 (29%)	582 (29%)	
Alcohol%					0.001
Non-drinker	602 (20%)	543 (19%)	538 (18%)	536 (20%)	
1–5 drinks/month	1,057 (47%)	1,102 (48%)	1,082 (49%)	1,038 (45%)	
5–10 drinks/month	168 (8.5%)	212 (10%)	183 (9.1%)	151 (7.9%)	
10+ drinks/month	352 (17%)	346 (18%)	408 (20%)	466 (22%)	
Wait	130 (7.5%)	106 (4.8%)	98 (3.8%)	118 (4.9%)	
Comorbidities (YES%)					
Liver disease	117 (4.4%)	93 (4.0%)	114 (5.0%)	131 (5.0%)	0.551
Arthritis	753 (30%)	826 (33%)	867 (35%)	1,017 (42%)	<0.001
COPD	1,216 (53%)	1,286 (55%)	1,318 (55%)	1,606 (69%)	<0.001
Asthma	1,504 (64%)	1,463 (63%)	1,396 (61%)	1,160 (51%)	<0.001
Diabetes					<0.001
Normal	1,278 (61%)	1,272 (61%)	1,212 (58%)	1,075 (50%)	
Prediabetes	645 (27%)	663 (26%)	662 (28%)	702 (31%)	
Diabetes	386 (12%)	374 (12%)	435 (14%)	532 (19%)	
Hypertension	850 (32%)	842 (33%)	948 (36%)	1,114 (45%)	<0.001
Cancer	167 (8.3%)	212 (10.0%)	286 (13%)	408 (17%)	<0.001
HF^c^	74 (2.6%)	87 (2.8%)	130 (4.5%)	270 (9.8%)	<0.001
HLP^c^	1,165 (51%)	1,176 (51%)	1,181 (51%)	1,246 (54%)	0.41
CKD^c^	61 (2.0%)	64 (2.1%)	77 (2.6%)	158 (5.0%)	<0.001
CHD^c^	90 (3.5%)	100 (4.1%)	149 (5.0%)	245 (9.2%)	<0.001
Laboratory test					
LDH U/L	127 (112, 143)	126 (112, 142)	129 (114, 144)	132 (118, 150)	<0.001
AST U/L	21 (16, 28)	21 (17, 29)	21 (16, 29)	20 (16, 28)	0.087
ALT U/L	23 (19, 27)	23 (19, 27)	23 (19, 27)	23 (19, 27)	0.844
HGB g/dL	14.30 (13.40, 15.10)	14.40 (13.50, 15.40)	14.50 (13.50, 15.40)	14.30 (13.10, 15.40)	<0.001
WBC 1000/uL	7.00 (5.70, 8.50)	7.20 (6.00, 8.60)	7.10 (5.90, 8.80)	7.60 (6.30, 9.30)	<0.001
PLT 1000/uL	260 (220, 303)	256 (220, 299)	256 (217, 299)	250 (209, 298)	0.005
ALB g/dL	4.30 (4.10, 4.50)	4.30 (4.10, 4.50)	4.30 (4.00, 4.50)	4.10 (3.90, 4.40)	<0.001
Cr mg/dL	0.80 (0.70, 0.94)	0.82 (0.70, 0.99)	0.84 (0.70, 1.00)	0.90 (0.72, 1.02)	<0.001
Bun mg/dL	12.0 (9.0, 14.0)	12.0 (10.0, 15.0)	12.0 (10.0, 16.0)	13.0 (10.0, 17.0)	<0.001
Ga mg/dL	9.50 (9.30, 9.70)	9.50 (9.20, 9.70)	9.40 (9.20, 9.70)	9.40 (9.20, 9.60)	<0.001
Na mmol/L	139.0 (138.0, 141.0)	139.0 (138.0, 141.0)	139.0 (138.0, 140.9)	139.0 (137.0, 141.0)	0.149
K mmol/L	4.00 (3.80, 4.20)	4.00 (3.80, 4.20)	4.00 (3.80, 4.20)	4.00 (3.80, 4.30)	<0.001
TBIL umol/L	0.60 (0.50, 0.80)	0.70 (0.50, 0.80)	0.70 (0.50, 0.80)	0.70 (0.50, 0.80)	0.003
OSM3 mmol/kg	278.0 (275.0, 280.0)	278.0 (275.0, 281.0)	278.0 (275.0, 281.0)	278.0 (275.0, 282.0)	0.002
CRP mg/dL	0.20 (0.09, 0.41)	0.21 (0.09, 0.44)	0.24 (0.10, 0.49)	0.30 (0.15, 0.65)	<0.001
Novel inflammatory marker					
NLR^d^	1.33 (1.06, 1.69)	1.83 (1.57, 2.17)	2.29 (1.94, 2.71)	3.25 (2.67, 4.13)	<0.001
MLR^d^	0.18 (0.15, 0.20)	0.24 (0.21, 0.26)	0.30 (0.27, 0.33)	0.41 (0.36, 0.50)	<0.001
SIRI^d^	0.62 (0.46, 0.81)	0.97 (0.80, 1.18)	1.31 (1.06, 1.61)	2.14 (1.68, 2.83)	<0.001
lgSII^d^	5.86 (5.56, 6.13)	6.17 (5.92, 6.41)	6.37 (6.12, 6.62)	6.72 (6.44, 7.00)	<0.001
lgPLR^d^	4.59 (4.37, 4.80)	4.75 (4.56, 4.94)	4.89 (4.68, 5.10)	5.09 (4.87, 5.32)	<0.001
NAPR^d^	11.76 (10.43, 13.11)	13.37 (12.20, 14.59)	14.37 (13.24, 15.64)	16.03 (14.81, 17.80)	<0.001
Outcome					
Follow-up time (months)	139 (96, 193)	144 (98, 188)	136 (94, 188)	119 (75, 174)	<0.001
CLRD-specific mortality%	32 (1.0%)	34 (1.2%)	71 (2.7%)	130 (5.4%)	<0.001
All-cause mortality%	317 (11%)	414 (15%)	501 (17%)	933 (33%)	<0.001

^a^ n (unweighted) (%); Median (IQR).

^b^ chi-squared test with Rao & Scott’s second-order correction; Wilcoxon rank-sum test for complex survey samples.

^c^ HF: heart failure, HLP: hyperlipidemia, CKD: chronic kidney disease, CHD: coronary heart disease, OSM:serum osmolality.

^d^ NLR: neutrophil-to-lymphocyte ratio, MLR:monocyte-to-lymphocyte ratio, SIRI:systemic inflammation response index, lgSII:logarithmic systemic immune-inflammation index, lgPLR:logarithmic platelet-to-lymphocyte ratio, NPAR: neutrophil percentage to albumin ratio. NMLAR: (Neutrophil% × Monocyte% × 100)/(Lymphocyte% × Albumin [g/dL]).

### ROC curve analysis

**FIGURE 3:**
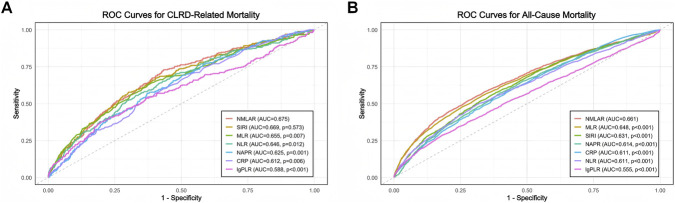
Comparison of the discriminatory ability of NMLAR and other inflammatory markers using ROC curves for mortality outcomes. **(A)** ROC curves for CLRD-specific mortality. **(B)** ROC curves for all-cause mortality. NMLAR demonstrated the highest area under the curve (AUC) among all tested markers in both outcomes (AUC = 0.675 for CLRD-specific mortality; AUC = 0.661 for all-cause mortality). Other markers compared include SIRI, MLR, NLR, NAPR, CRP, and lgPLR. P values were calculated using DeLong’s test for comparing AUCs between NMLAR and each of the other markers.

In predicting CLRD-specific mortality (Figure 3A), NMLAR achieved the highest area under the curve (AUC = 0.675), slightly outperforming SIRI (AUC = 0.669, p = 0.573), MLR (AUC = 0.655, p = 0.007), and NLR (AUC = 0.646, p = 0.012), and demonstrating significantly better discrimination than NAPR (AUC = 0.625, p = 0.001), CRP (AUC = 0.612, p = 0.006), and lgPLR (AUC = 0.588, p < 0.001). Similarly, in predicting all-cause mortality (Figure 3B), NMLAR yielded the highest AUC (0.661), exceeding those of MLR (0.648), SIRI (0.631), NAPR (0.614), CRP (0.611), NLR (0.611), and lgPLR (0.555), all with p-values <0.001. These findings indicate that NMLAR demonstrates superior discriminative ability compared with most other inflammatory markers for both CLRD-specific and all-cause mortality.

### Feature selection

**FIGURE 4:**
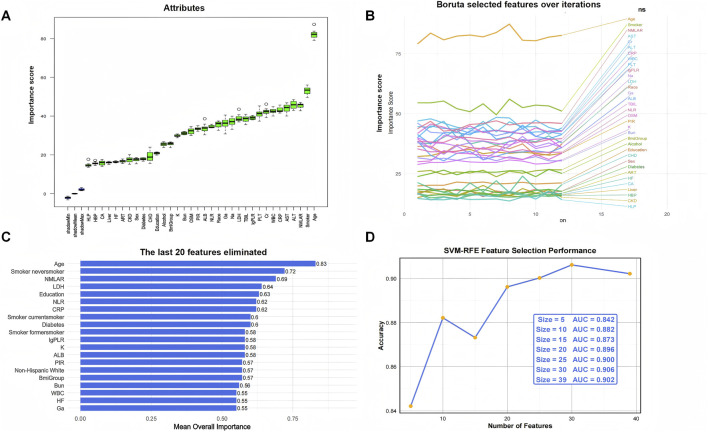
Feature selection results for CLRD-specific mortality using Boruta and SVM-RFE algorithms. **(A)** Importance ranking of features by Boruta, where green boxplots represent features classified as “confirmed important.” NMLAR ranked third in terms of mean Z-score importance, following age and smoking status. **(B)** Trajectory plot showing the stability of feature importance scores over 20 iterations in Boruta. The top three consistently important features—age, smoking status, and NMLAR—are highlighted in orange. **(C)** The last 20 features eliminated by SVM-RFE, ranked by overall importance. NMLAR was the third-to-last feature eliminated, suggesting high predictive relevance. **(D)** SVM-RFE performance curve showing AUC values corresponding to different numbers of retained features. The model achieved an AUC of 0.882 with the top 10 features, with marginal gains beyond this point, supporting the selection of 10 variables for final modeling.

**FIGURE 5:**
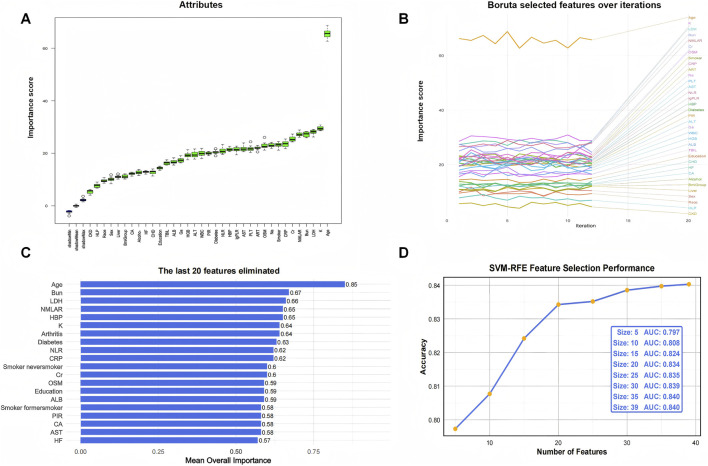
Feature selection results for all-cause mortality using Boruta and SVM-RFE algorithms. **(A)** Feature importance ranking based on the Boruta algorithm. Green boxplots indicate features confirmed as important, with NMLAR ranking fifth in mean Z-score importance. **(B)** Feature stability plot across 20 Boruta iterations, highlighting age, BUN, LDH, and NMLAR as consistently top-ranked features. **(C)** The last 20 features eliminated in SVM-RFE, where NMLAR was the fourth-to-last feature removed, suggesting strong predictive value. **(D)** SVM-RFE performance curve showing AUC values for different numbers of selected features. AUC increased with more variables and plateaued beyond 30 features, reaching 0.840. The top 10 variables (AUC = 0.808) were retained in the final model to balance performance and simplicity.

**FIGURE 6:**
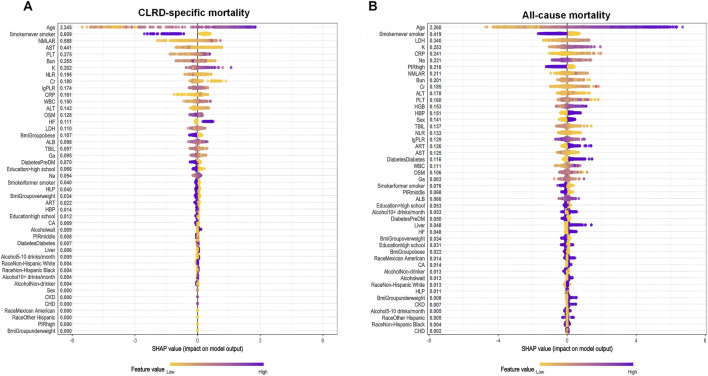
SHAP summary plots derived from the XGBoost model, illustrating the impact of individual features on the prediction of CLRD-specific and all-cause mortality. **(A)**For CLRD-specific mortality, the most influential predictors were age, smoking status (never smoker), and NMLAR, with higher SHAP values indicating greater impact on model predictions. **(B)**For all-cause mortality, age remained the most impactful variable, followed by smoking status, LDH, and CRP. Notably, NMLAR ranked 8th in mean SHAP value among all predictors for all-cause mortality. Each point represents the SHAP value of a feature for a single individual, colored by the feature value (yellow = low, purple = high). Features are ranked by their mean absolute SHAP value. SHAP values were calculated based on the final XGBoost model to enhance model interpretability.

Given the complex survey design of the NHANES database, we first applied weighted resampling to account for sampling weights prior to conducting machine learning analyses. Three established algorithms—Boruta, SVM-RFE, and XGBoost—were employed to identify the most relevant predictors for CLRD-specific and all-cause mortality. NMLAR consistently ranked as the third most important feature for CLRD-specific mortality across all three machine learning algorithms (Figure 4). Specifically, it ranked third based on mean Z-score importance in the Boruta algorithm (Figure 4A; Supplementary Figure S2), was the third-to-last feature eliminated in SVM-RFE (Figure 4C), and also held the third position according to both SHAP value and Gain-based importance in the XGBoost model (Figure 6A; Supplementary Figure S3)—consistently following only age and smoking across all methods. For all-cause mortality (Figure 5), NMLAR also demonstrated high importance, ranking among the top predictors across all three algorithms—fifth by Z-score (Figure 5A; Supplementary Figure S4), fourth-to-last in SVM-RFE (Figure 5C), and within the top eight by both SHAP value and Gain in the XGBoost model (Figure 6B; Supplementary Figure S5). These findings indicate that NMLAR is one of the key contributors to model output across diverse analytical frameworks. Feature selection based on SVM-RFE was performed separately for CLRD-specific and all-cause mortality. As shown in the performance curves, for CLRD-specific mortality (Figure 4D), the model reached an AUC of 0.882 when the top 10 features were retained, with only marginal improvements observed beyond this point (peak AUC = 0.906 at 30 features). For all-cause mortality (Figure 5D), the AUC increased modestly from 0.797 (5 features) to 0.808 (10 features), with limited gains afterward (maximum AUC = 0.840). Considering the trade-off between model performance and complexity, we retained the top 10 variables in both models for subsequent analyses. The top 10 features identified by each algorithm were merged to form the covariate set used in Model 3. For the CLRD-specific mortality model, the final covariates included: age, smoking status, NMLAR, diabetes, LDH, AST, ALT, WBC, PLT, Cr, BUN, K, CRP, NLR, lgPLR, and education level. For the all-cause mortality model, selected variables included: age, LDH, PLT, K, hypertension, NMLAR, BUN, CRP, Cr, NLR, lgPLR, diabetes, arthritis, smoking status, and Na.

### Cox regression analysis

In the Cox proportional hazards models, NMLAR remained significantly associated with both CLRD-specific and all-cause mortality across all levels of model adjustment (Table 2). In the fully adjusted model (Model 3), which accounted for covariates selected via machine learning, the HRs decreased slightly with deeper adjustment but retained statistical significance. When modeled as a categorical variable, participants in the highest NMLAR quartile (Q4) had a significantly elevated risk of CLRD-specific mortality compared to the lowest quartile (Q1) (HR = 2.55, 95% CI: 1.21–5.36). Similarly, Q4 was associated with increased all-cause mortality (HR = 1.62, 95% CI: 1.28–2.06). When treated as a continuous variable, each one-unit increase in NMLAR was associated with a 7% increase in CLRD-specific mortality risk (HR = 1.07, 95% CI: 1.01–1.14) and an 8% increase in all-cause mortality risk (HR = 1.08, 95% CI: 1.06–1.11). Stratified analyses by disease subtype demonstrated consistent positive associations. Among individuals with generalized COPD (including emphysema and chronic bronchitis), those in Q4 had markedly higher risks of CLRD-specific mortality (HR = 3.16, 95% CI: 1.34–7.49) and all-cause mortality (HR = 1.52, 95% CI: 1.17–1.96). In the asthma subgroup, Q4 was associated with elevated CLRD-specific mortality (HR = 2.26, 95% CI: 0.84–6.09) and all-cause mortality (HR = 1.79, 95% CI: 1.27–2.50). As a continuous predictor, NMLAR was associated with a 7% increase in CLRD-specific mortality (HR = 1.07, 95% CI: 1.01–1.14) and a 9% increase in all-cause mortality (HR = 1.09, 95% CI: 1.06–1.12) among the COPD subgroup. In the asthma subgroup, the corresponding HRs were 1.11 (95% CI: 1.02–1.20) for CLRD-specific mortality and 1.10 (95% CI: 1.06–1.14) for all-cause mortality. These findings underscore the robust and independent prognostic value of NMLAR across different CLRD phenotypes. In addition, trend tests across NMLAR quartiles were statistically significant for both CLRD-specific and all-cause mortality in all models and subgroups, further supporting a consistent dose–response association.

**TABLE 2:** Cox regression analysis of NMLAR and mortality.

Model	Continuous	Quartile 1	Quartile 2	Quartile 3	Quartile 4	p for trend
CLRD-specific mortality						
Model 1	1.22 (1.18–1.26) <0.001	Reference	1.21 (0.66–2.24) 0.536	2.73 (1.60–4.65) <0.001	6.33 (3.88–10.34) <0.001	<0.001
Model 2	1.12 (1.09–1.16) <0.001	Reference	1.02 (0.56–1.85) 0.961	1.73 (1.02–2.93) 0.043	2.89 (1.80–4.66) <0.001	<0.001
Model 3	1.07 (1.01–1.14) 0.021	Reference	1.09 (0.59–2.03) 0.776	1.86 (1.02–3.37) 0.042	2.55 (1.21–5.36) 0.014	0.003
CLRD-specific mortality in COPD						
Model 1	1.19 (1.15–1.23) <0.001	Reference	1.62 (0.78–3.37) 0.195	3.67 (2.07–6.52) <0.001	7.32 (4.06–13.21) <0.001	<0.001
Model 2	1.12 (1.09–1.16) <0.001	Reference	1.41 (0.71–2.81) 0.322	2.51 (1.42–4.44) 0.001	3.82 (2.15–6.81) <0.001	<0.001
Model 3	1.07 (1.01–1.14) 0.027	Reference	1.54 (0.75–3.18) 0.238	2.67 (1.34–5.34) 0.005	3.16 (1.34–7.49) 0.009	0.002
CLRD-specific mortality in asthma						
Model 1	1.26 (1.20–1.32) <0.001	Reference	0.42 (0.14–1.22) 0.110	1.89 (0.93–3.82) 0.079	5.89 (3.00–11.56) <0.001	<0.001
Model 2	1.15 (1.09–1.21) <0.001	Reference	0.35 (0.12–1.04) 0.060	1.24 (0.58–2.64) 0.575	2.54 (1.24–5.19) 0.010	<0.001
Model 3	1.11 (1.02–1.20) 0.011	Reference	0.42 (0.14–1.27) 0.126	1.33 (0.55–3.19) 0.524	2.26 (0.84–6.09) 0.105	0.021
All-cause mortality						
Model 1	1.19 (1.16–1.21) <0.001	Reference	1.39 (1.13–1.70) 0.001	1.66 (1.36–2.02) <0.001	3.69 (3.06–4.45) <0.001	<0.001
Model 2	1.11 (1.08–1.13) <0.001	Reference	1.21 (0.99–1.49) 0.061	1.13 (0.93–1.38) 0.213	1.89 (1.56–2.29) <0.001	<0.001
Model 3	1.08 (1.06–1.11) <0.001	Reference	1.25 (1.00–1.56) 0.050	1.15 (0.92–1.43) 0.214	1.62 (1.28–2.06) <0.001	<0.001
All-cause mortality in COPD						
Model 1	1.16 (1.14–1.19) <0.001	Reference	1.18 (0.94–1.47) 0.145	1.58 (1.30–1.93) <0.001	3.30 (2.75–3.95) <0.001	<0.001
Model 2	1.09 (1.07–1.11) <0.001	Reference	1.03 (0.84–1.27) 0.780	1.10 (0.90–1.35) 0.362	1.73 (1.41–2.13) <0.001	<0.001
Model 3	1.09 (1.06–1.12) <0.001	Reference	1.05 (0.84–1.30) 0.667	1.11 (0.88–1.39) 0.381	1.52 (1.17–1.96) <0.001	<0.001
All-cause mortality in asthma						
Model 1	1.21 (1.18–1.24) <0.001	Reference	1.35 (1.01–1.80) 0.044	1.64 (1.26–2.14) <0.001	3.60 (2.83–4.59) <0.001	<0.001
Model 2	1.12 (1.09–1.14) <0.001	Reference	1.21 (0.88–1.65) 0.236	1.27 (0.92–1.75) 0.141	1.95 (1.46–2.60) <0.001	<0.001
Model 3	1.10 (1.06–1.14) <0.001	Reference	1.38 (1.01–1.88) 0.045	1.37 (0.99–1.90) 0.061	1.79 (1.27–2.50) <0.001	<0.001

Model 1 was unadjusted.

Model 2 was adjusted for age and sex.

Model 3 was further adjusted for key predictors identified by three machine learning methods. For the CLRD-specific mortality model, covariates included age, smoking status, NMLAR, diabetes, LDH, AST, ALT, WBC, PLT, cr, BUN, K, CRP, NLR, lgPLR, and education level. For the all-cause mortality model, covariates included age, LDH, PLT, K, hypertension, NMLAR, BUN, CRP, cr; NLR, lgPLR, diabetes, arthritis, smoking status.

### Restricted cubic spline analysis

**FIGURE 7:**
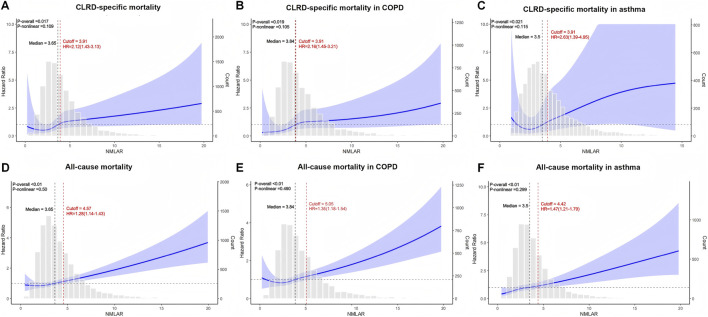
Restricted cubic spline (RCS) analysis of the association between NMLAR and mortality outcomes based on Model 3. **(A–C)** RCS curves for CLRD-specific mortality in the overall population **(A)**, COPD subgroup **(B)**, and asthma subgroup **(C)**. **(D–F)** RCS curves for all-cause mortality in the overall population **(D)**, COPD subgroup **(E)**, and asthma subgroup **(F)**. The blue lines represent the adjusted hazard ratios (HRs) for mortality across continuous values of NMLAR, with shaded areas indicating 95% confidence intervals. The red dashed lines indicate the optimal cutoff values identified using the maximum Youden index, along with corresponding HRs and 95% confidence intervals for values above the cutoff. P values for the overall association and non-linearity are provided in the top left corner of each plot. Gray bars show the distribution of NMLAR. All models were adjusted for covariates included in Model 3. cutoff = Youden index.

RCS analyses revealed a linear and monotonically increasing association between NMLAR and both CLRD-specific and all-cause mortality, with no apparent nonlinear inflection points or plateau phases. This linear trend was consistently observed in the overall population as well as in the generalized COPD and asthma subgroups. For CLRD-specific mortality, the optimal Youden index cutoff was 3.91 across the total cohort, the COPD subgroup, and the asthma subgroup. Based on this threshold, individuals were stratified into high- and low-risk groups. Compared with the low-risk group, the high-risk group demonstrated significantly increased risks of CLRD-specific death: overall population HR = 2.12 (95% CI: 1.51–2.96); COPD subgroup HR = 2.16 (95% CI: 1.34–3.47); asthma subgroup HR = 2.63 (95% CI: 1.36–5.06). For all-cause mortality, the optimal Youden index was 4.57 in the overall population, 5.05 in the generalized COPD subgroup, and 4.42 in the asthma subgroup. Stratified analyses indicated that high-risk individuals had significantly greater all-cause mortality: overall HR = 1.28 (95% CI: 1.13–1.44); COPD subgroup HR = 1.35 (95% CI: 1.15–1.59); asthma subgroup HR = 1.47 (95% CI: 1.22–1.76). These findings further underscore the prognostic utility of NMLAR as a robust, continuous inflammatory biomarker for mortality risk stratification across diverse CLRD phenotypes (Figure 7).

### Subgroup Analysis

**FIGURE 8:**
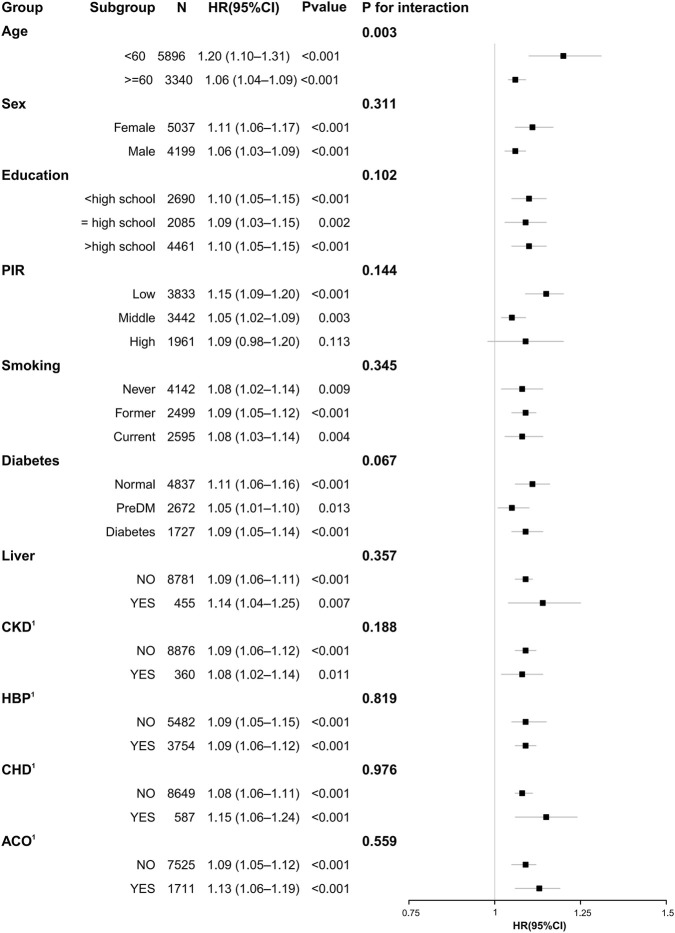
Subgroup analysis of the association between NMLAR and all-cause mortality based on Model 3.

**FIGURE 9:**
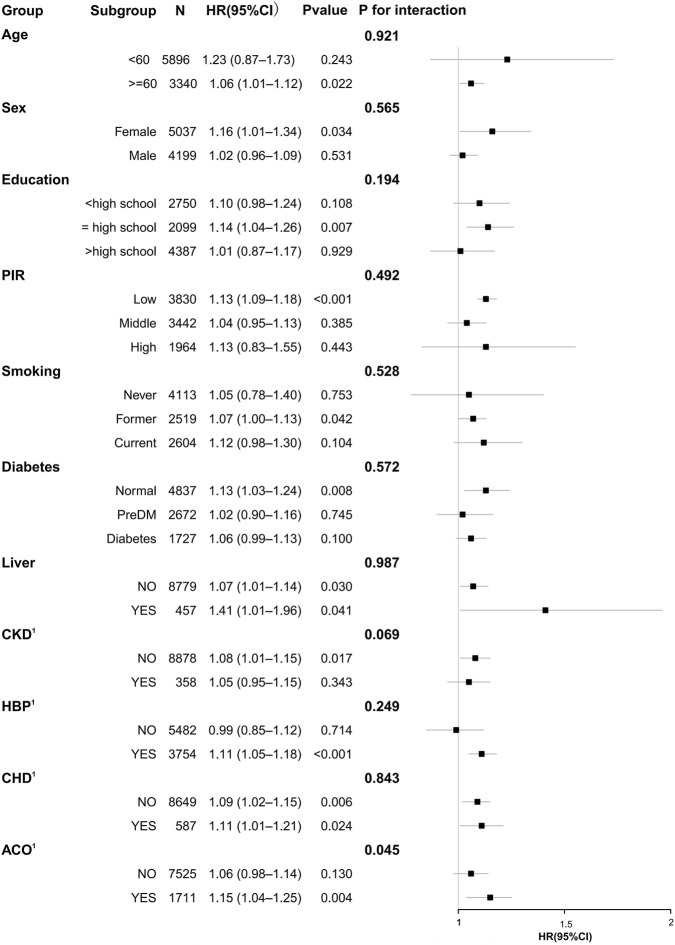
Subgroup analysis of the association between NMLAR and CLRD-specific mortality based on Model 3.

Subgroup analyses across demographic characteristics, socioeconomic status, smoking status, and comorbidities demonstrated that elevated NMLAR was consistently associated with increased mortality risk. For all-cause mortality (Figure 8), the association was evident in both younger (<60 years: HR = 1.20, 95% CI: 1.10–1.31) and older participants (≥60 years: HR = 1.06, 95% CI: 1.04–1.09), with a significant interaction for age (P for interaction = 0.003), indicating a stronger effect in the younger population; results were generally stable across sex, education, PIR, smoking, and comorbidity subgroups including diabetes, liver disease, CKD, hypertension, CHD, and ACO (yes = asthma–COPD overlap; no = asthma or COPD alone). For CLRD-specific mortality (Figure 9), significant associations were observed in women, participants with high school education, low PIR, non-diabetic individuals, those without liver disease as well as those with liver disease, and in participants with hypertension or CHD, with hazard ratios ranging from 1.07 to 1.41. Importantly, a significant interaction was detected for ACO (P for interaction = 0.045), with a stronger association in the asthma–COPD overlap subgroup (HR = 1.15, 95% CI: 1.04–1.25) compared with asthma or COPD alone, while no other interactions reached significance.

### Model performance evaluation

**FIGURE 10:**
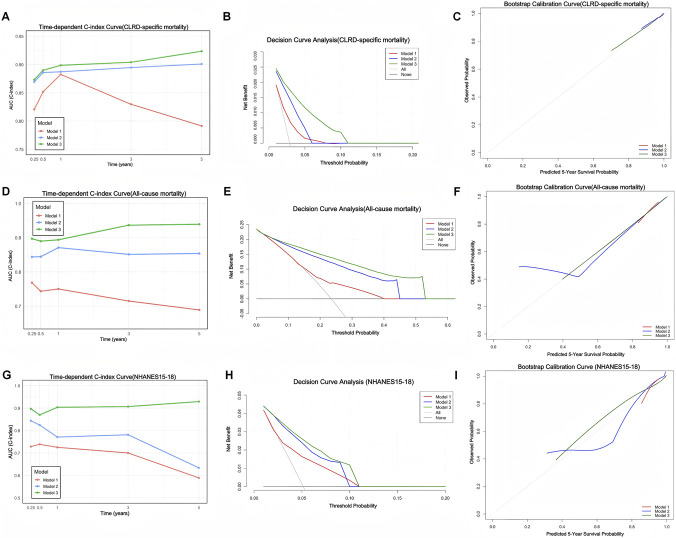
Evaluation of model performance across three prognostic models for CLRD-specific mortality, all-cause mortality, and external validation using NHANES 2015–2018. **(A,D,G)** Time-dependent concordance index (C-index) curves showing the discrimination ability of Model 1 (red), Model 2 (blue), and Model 3 (green) over time for CLRD-specific mortality **(A)**, all-cause mortality **(D)**, and the external validation cohort **(G)**. **(B,E,H)** Decision curve analysis (DCA) plots illustrating the clinical net benefit of each model across a range of threshold probabilities in the same populations. **(C,F,I)** Bootstrap calibration curves (B = 1000) at the 5-year time point evaluating agreement between predicted and observed survival probabilities for each model in the corresponding cohorts.

We comprehensively evaluated the performance of three Cox proportional hazards models in predicting both CLRD-specific and all-cause mortality, using time-dependent C-index, DCA, and bootstrap-based calibration curves. In the training cohort, Model 3 demonstrated consistently superior predictive performance compared to Model 1 and Model 2. As shown in Figure 10, Model 3 achieved the highest C-index throughout the follow-up period for both outcomes. Specifically, for CLRD-specific mortality, the C-index at 3 months, 6 months, 1 year, 3 years, and 5 years was 0.87, 0.89, 0.90, 0.90, and 0.92 (Figure 10A), respectively. For all-cause mortality, corresponding values were 0.90, 0.89, 0.90, 0.94, and 0.94 (Figure 10D). DCA curves further confirmed that Model 3 provided the greatest net clinical benefit in mortality prediction (Figures 10B,E). Bootstrap calibration plots demonstrated that Model 3 exhibited the best model calibration and goodness-of-fit among all models (Figures 10C,F). In the external validation cohort from NHANES 2015–2018, Model 3 maintained strong generalizability. In predicting all-cause mortality, Model 3 consistently achieved the highest C-index, reaching 0.93 at 5 years of follow-up (Figure 10G). DCA analysis in the validation set again showed superior net clinical benefit for Model 3 (Figure 10H), and calibration plots confirmed the model’s reliability and consistency (Figure 10I).

### External validation

**FIGURE 11:**
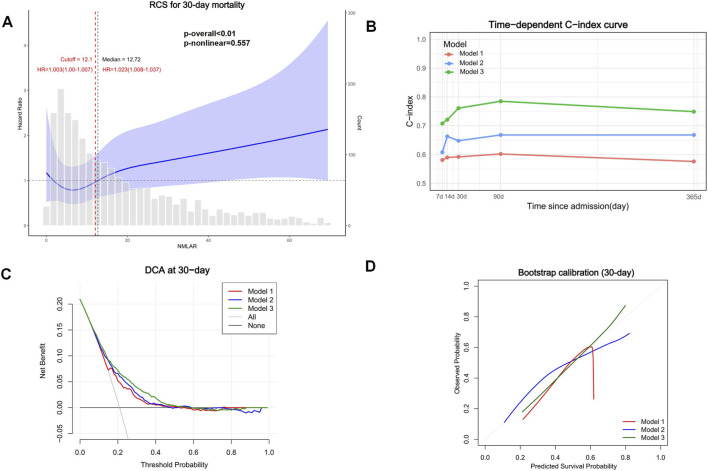
External validation of NHANES models in the MIMIC cohort (30-day mortality). RCS showed an approximately linear association between NMLAR and mortality, consistent with NHANES **(A)**. Model 3 demonstrated moderate discrimination with C-indices of 0.708, 0.721, 0.761, 0.785, and 0.740 at 7, 14, 30, 90, and 365 days **(B)**, respectively, along with good calibration and greater clinical net benefit **(C)** on decision-curve analysis **(D)**.

In the MIMIC cohort, 2,120 patients were included. Crude all-cause mortality at 7, 14, 30, 90, and 365 days was 8.6%, 13.0%, 16.0%, 21.0%, and 31.0%, respectively. The baseline table demonstrated a monotonic dose–response: across increasing NMLAR quartiles (Q1-Q4), mortality rose for all endpoints (Supplementary Table S2). Consistent with NHANES, higher NMLAR was positively associated with subsequent mortality and remained statistically significant after progressive adjustment at 14–365 days. When modeled continuously, Model 3 showed that per-unit increases in NMLAR were associated with significantly higher mortality at 14 days (HR 1.042, 95% CI 1.010–1.075, p < 0.01), 30 days (HR 1.048, 95% CI 1.012–1.086, p < 0.01), 90 days (HR 1.044, 95% CI 1.011–1.078, p < 0.01), and 365 days (HR 1.040, 95% CI 1.007–1.074, p = 0.016). In contrast, the 7-day association was nonsignificant (HR 1.034, p = 0.529). In quartile analyses (Q1 reference), Model 3 estimated Q4 vs. Q1 HRs (95% CIs) of 2.257 (1.313–3.882) at 14 days, 2.075 (1.284–3.354) at 30 days, and 1.558 (1.042–2.331) at 90 days (all significant); the 365-day comparison was borderline (1.353 [0.970–1.889], p = 0.075), and the 7-day comparison was not significant (1.638 [0.839–3.201], p = 0.149). P for trend across quartiles was <0.01 at all horizons, indicating increasing risk with higher NMLAR (Table 3). In external validation by transporting the NHANES models to MIMIC without refitting, Model 3 achieved C-indices of 0.708, 0.721, 0.761, 0.785, and 0.740 at 7, 14, 30, 90, and 365 days, respectively—consistent with moderate discrimination—with good calibration and greater clinical net benefit on decision-curve analysis. RCS mirrored NHANES, indicating an approximately linear association between NMLAR and mortality in MIMIC inpatients. Notably, compared with the NHANES general population, the NMLAR distribution in MIMIC was broader and right-shifted, reflecting a higher central tendency and greater dispersion. Threshold analysis further identified a cutoff at 12.10: below this value, each 1-unit increase in NMLAR was not associated with a significant change in mortality risk (HR = 1.003, 95% CI: 1.000–1.007), whereas above this threshold, the risk increased significantly (HR = 1.023, 95% CI: 1.008–1.037) (Figure 11; Supplementary Figure S6). Subgroup analyses yielded consistent results across all predefined strata (Supplementary Table S3).

**TABLE 3:** Cox regression analysis of NMLAR and mortality in external cohort.

Model	Continuous	Quartile 1	Quartile 2	Quartile 3	Quartile 4	P For trend
7-day mortality from MIMIC						
Model 1	1.012 (1.003–1.022) <0.01	Reference	1.444 (0.756–2.756) 0.265	1.706 (0.931–3.126) 0.083	2.268 (1.253–4.105) <0.01	<0.01
Model 2	1.004 (0.993–1.015) 0.486	Reference	1.417 (0.729–2.754) 0.304	1.434 (0.752–2.736) 0.274	1.658 (0.845–3.255) 0.142	<0.01
Model 3	1.004 (0.992–1.015) 0.529	Reference	1.444 (0.745–2.800) 0.277	1.461 (0.768–2.778) 0.248	1.638 (0.839–3.201) 0.149	<0.01
14-day mortality from MIMIC						
Model 1	1.018 (1.011–1.025) <0.01	Reference	1.355 (0.818–2.245) 0.238	1.671 (1.040–2.685) 0.034	2.595 (1.644–4.095) <0.01	<0.01
Model 2	1.013 (1.004–1.022) <0.01	Reference	1.550 (0.910–2.640) 0.107	1.672 (0.983–2.842) 0.058	2.279 (1.320–3.936) <0.01	<0.01
Model 3	1.013 (1.004–1.022) <0.01	Reference	1.563 (0.921–2.654) 0.098	1.654 (0.977–2.802) 0.061	2.257 (1.313–3.882) <0.01	<0.01
30-day mortality from MIMIC						
Model 1	1.020 (1.014–1.027) <0.01	Reference	1.163 (0.736–1.837) 0.518	1.644 (1.081–2.501) 0.020	2.506 (1.668–3.766) <0.01	<0.01
Model 2	1.015 (1.007–1.023) <0.01	Reference	1.218 (0.754–1.965) 0.420	1.516 (0.954–2.409) 0.079	2.086 (1.288–3.378) <0.01	<0.01
Model 3	1.015 (1.007–1.022) <0.01	Reference	1.241 (0.770–2.001) 0.375	1.527 (0.962–2.422) 0.073	2.075 (1.284–3.354) 0.003	<0.01
90-day mortality from MIMIC						
Model 1	1.014 (1.008–1.020) <0.01	Reference	1.121 (0.770–1.633) 0.551	1.385 (0.977–1.964) 0.067	1.874 (1.330–2.641) <0.01	<0.01
Model 2	1.010 (1.002–1.016) 0.016	Reference	1.131 (0.763–1.675) 0.536	1.259 (0.857–1.850) 0.240	1.541 (1.029–2.307) 0.036	<0.01
Model 3	1.009 (1.002–1.015) 0.015	Reference	1.157 (0.782–1.713) 0.466	1.275 (0.869–1.871) 0.215	1.558 (1.042–2.331) 0.031	<0.01
365-day mortality from MIMIC						
Model 1	1.010 (1.005–1.015) <0.01	Reference	1.009 (0.742–1.373) 0.953	1.188 (0.892–1.583) 0.238	1.511 (1.137–2.007) <0.01	<0.01
Model 2	1.007 (1.000–1.012) 0.034	Reference	1.022 (0.741–1.409) 0.896	1.109 (0.810–1.519) 0.519	1.336 (0.956–1.866) 0.088	<0.01
Model 3	1.006 (1.001–1.012) 0.030	Reference	1.045 (0.758–1.441) 0.789	1.115 (0.814–1.526) 0.497	1.353 (0.970–1.889) 0.075	<0.01
30-day mortality from zhejiang provencial ICU						
Model 1	1.139 (1.063–1.221) <0.01	Reference	2.050 (0.776–5.415) 0.147	4.366 (1.892–10.083) <0.01	5.837 (2.234–15.007) <0.01	<0.01
Model 2	1.136 (1.060–1.220) <0.01	Reference	1.899 (0.717–5.309) 0.196	4.109 (1.810–9.300) <0.01	5.231 (2.015–13.579) <0.001	<0.01
Model 3	1.093 (1.012–1.180) <0.01	Reference	1.400 (0.508–3.872) 0.589	3.116 (1.814–5.360) <0.01	4.556 (1.480–11.007) <0.001	<0.01

MIMIC: Model 1: Unadjusted. Model 2: Adjusted for key predictors identified in NHANES, including age, LDH, PLT, K, hypertension, NMLAR, BUN, CRP, creatinine; NLR, lgPLR, diabetes, arthritis, smoking status. Model 3: Further adjusted for Model 2 covariates plus MIMIC-specific clinical factors, including SOFA, score, use of CRRT, vasopressor use, and mechanical ventilation. EHR:Model 1 was unadjusted. Model 2 was adjusted for age and sex. Model 3 was further adjusted for age, LDH, PLT, hypertension, NMLAR, BUN, CRP, cr; NLR, lgPLR, diabetes, arthritis.

**FIGURE 12:**
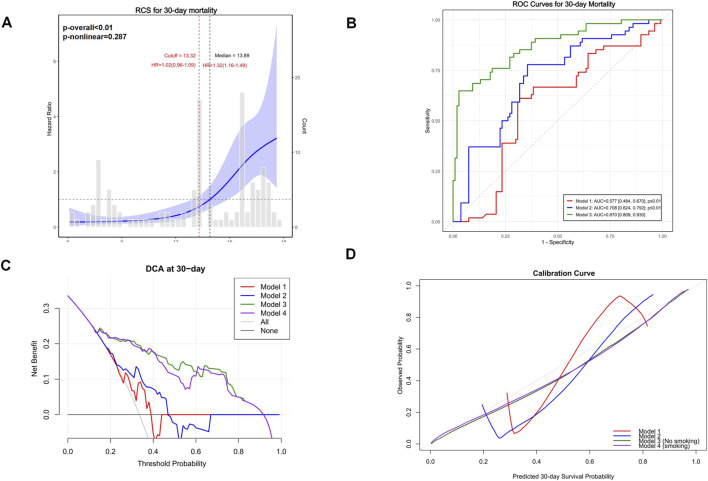
External validation of NHANES models in the Zhejiang Provincial ICU cohort (30-day mortality). RCS revealed an approximately linear association between NMLAR and 30-day mortality, with a threshold identified at NMLAR = 13.32, above which the risk increased markedly **(A)**. ROC curves demonstrated that Model 3 achieved the highest discrimination (AUC = 0.879, 95% CI: 0.809–0.930), outperforming Model 1 (AUC = 0.577) and Model 2 (AUC = 0.702) **(B)**. Decision-curve analysis further confirmed greater net clinical benefit for Models 3 and 4, where Model 3 assumed all patients were nonsmokers and Model 4 assumed all patients were smokers **(C)**. Calibration curves indicated good agreement between predicted and observed probabilities under both assumptions **(D)**.

In the Zhejiang Provincial ICU cohort (n = 161), higher NMLAR levels were significantly associated with increased 30-day mortality, with HRs per unit increase of 1.139 (95% CI: 1.063–1.221), 1.136 (95% CI: 1.060–1.220), and 1.093 (95% CI: 1.012–1.180) in Models 1–3 (all p < 0.01) (Table 3; Supplementary Table S4). Restricted cubic spline analysis confirmed a linear association (p-overall <0.01; p-nonlinear = 0.287), with a threshold identified at NMLAR = 13.32, above which mortality risk increased sharply. Model discrimination improved progressively, with AUCs of 0.777, 0.802, and 0.879 for Models 1–3, respectively. Decision curve and calibration analyses further demonstrated that the fully adjusted model provided the greatest net clinical benefit and the best agreement between predicted and observed 30-day mortality (Figure 12).

## Discussion

Immune-infiltration analyses across multiple human cohorts and experimental models revealed a consistent pattern in both disease and acute-exacerbation states, characterized by increased proportions of neutrophils and monocyte-lineage cells and reduced proportions of lymphocytes. Building on this robust immune signature, we mapped the transcriptomic deconvolution pattern—marked by innate immune enrichment and adaptive lymphocyte depletion—onto clinically accessible but long-overlooked relative immune-cell proportions in peripheral blood, and accordingly developed a composite prognostic index that integrates the relative neutrophil–monocyte-to-lymphocyte ratio with serum albumin (NMLAR). Its generalizability and clinical utility were subsequently validated across multiple population-based and critically ill cohorts.

In this nationally representative study based on the NHANES 1999–2018 cycles, we were the first to propose and systematically evaluate a novel granulocyte ratio–based inflammatory biomarker—NMLAR. Previous studies have shown that granulocyte alterations are key features of CLRD exacerbation (Lopez et al., 2023), and markers such as NLR and PLR are cost-effective tools for identifying high-risk COPD patients prone to frequent exacerbations in primary care settings (Fu et al., 2025). Although these novel inflammation-related indices involving neutrophils and lymphocytes have demonstrated promising predictive value across multiple disease states, the considerable heterogeneity and complex inflammatory pathways involved in CLRDs necessitate more accurate biomarkers to inform clinical intervention (Wang et al., 2021). Monocyte–macrophage populations play a central role in restraining pulmonary fibrosis, and elevated monocyte levels may reflect the progression and exacerbation of lower respiratory tract diseases (Ogger et al., 2020). In COPD lung tissue, enrichment of pro-inflammatory macrophage/monocyte subsets is believed to contribute significantly to the persistence of chronic inflammation (Hu et al., 2023). Conversely, in asthma, increased monocyte counts have been positively associated with disease severity, and reductions in monocytes have been shown to alleviate airway inflammation, improve clinical symptoms, and potentially slow lung function decline (Alavinezhad et al., 2022). These findings underscore the rationale for incorporating monocyte parameters into inflammatory indices in chronic respiratory disease research. Serum albumin, a major plasma protein involved in nutritional metabolism and immune modulation, has also been associated with adverse outcomes in chronic conditions. In COPD, hypoalbuminemia has been identified as an independent risk factor for pulmonary hypertension and poor prognosis (Zhou et al., 2024; Feng et al., 2023). Integrating albumin into prognostic indices is thus well-supported by prior evidence. For example, the neutrophil percentage to albumin ratio (NPAR) has been proposed as a dual-purpose biomarker for breast cancer risk and prognosis, aiding in early detection and personalized treatment planning (Su et al., 2025). NMLAR uniquely incorporates elevated neutrophil and monocyte levels—commonly observed in CLRD exacerbations (Xu et al., 2024)—into the numerator, while declining lymphocyte counts and hypoalbuminemia—reflective of impaired immune function and nutritional status—form the denominator. Unlike traditional metrics based on absolute granulocyte counts, our use of relative proportions allows for the dynamic capture of subtle inflammatory shifts. This ratio structure enhances the sensitivity and discriminatory capacity of systemic inflammatory markers. Building upon these insights, we further compared NMLAR with various established and emerging inflammatory indices using ROC curve analysis and DeLong tests. Results consistently demonstrated that NMLAR outperformed other markers in predicting both CLRD-specific and all-cause mortality, reinforcing its clinical utility.

In this study, we used three widely applied machine learning algorithms—Boruta, SVM-RFE, and XGBoost—to conduct comprehensive feature selection across all candidate variables (Liao et al., 2025). These algorithms are well suited for high-dimensional data and complex interactions and are increasingly used in epidemiologic and clinical prognostic modeling (Guan et al., 2024; Lee et al., 2022). For CLRD-specific mortality, NMLAR consistently ranked among the top three predictors across all algorithms—second only to age and smoking history—and showed substantially greater importance than traditional inflammatory markers such as NLR, PLR, and CRP, demonstrating strong robustness and explanatory power. NMLAR also ranked highly in all-cause mortality models, supporting its broad predictive value across different mortality outcomes. These findings highlight NMLAR as a promising composite inflammatory–immune biomarker for identifying high-risk individuals with CLRD. Age remained the strongest predictor for both mortality outcomes, consistent with previous prognostic research (Chen et al., 2025; Wang F. et al., 2025). Notably, fewer variables contributed meaningfully to CLRD-specific mortality than to all-cause mortality; SHAP plots showed that lower-ranked factors (e.g., alcohol use, CKD, CHD, sex, race) added minimal information to the CLRD-specific model, whereas contributions in the all-cause model were more diffuse. The relatively higher importance of NMLAR in CLRD-specific mortality models further suggests that systemic inflammation may play a more central role in disease-specific death among CLRD patients (Yousuf et al., 2022).

Importantly, this study did not evaluate NMLAR alone but incorporated it into Cox proportional hazards models informed by machine-learning–derived feature importance rankings. Using AUC to assess SVM-RFE feature subsets, we found that model discrimination remained stable when the top 10 predictors were retained, whereas further reduction led to a clear decline; therefore, these 10 features were selected as covariates. In the CLRD-specific mortality model, the highest-ranking variables included inflammation-related markers (NMLAR, WBC, NLR, lgPLR, CRP), hepatic indices (AST, ALT, LDH), renal markers (Cr, BUN), electrolytes (K), and smoking and diabetes. The predominance of inflammation–nutrition–immune variables suggests that systemic inflammation and nutritional status are central to CLRD-specific mortality. In contrast, the all-cause mortality model included a broader range of comorbidity-related variables (e.g., hypertension, arthritis), while still retaining renal, electrolyte, and inflammatory markers, reflecting the multifactorial etiology of all-cause mortality and the dilution of respiratory-specific mechanisms by non-respiratory causes. Consistent with prior evidence, hepatic dysfunction, renal impairment, and electrolyte disturbances are well-established mortality determinants across clinical cohorts (Liao et al., 2025; Wang F. et al., 2025; Lei et al., 2025). Taken together, the shared predictors—particularly inflammatory markers, renal indicators, and electrolytes—underscore their fundamental prognostic relevance across mortality outcomes, with NMLAR emerging as a strong and consistent predictor. The additional inflammatory and hepatic variables in the CLRD-specific model further indicate that inflammation–immune–nutritional dysregulation may play a more prominent role in disease-specific mortality (Peng et al., 2025).

In both CLRD-specific and all-cause mortality analyses, the HR for NMLAR decreased progressively with model adjustment but remained statistically significant, suggesting that NMLAR is an independent risk factor for both outcomes in the CLRD population. As a continuous variable, each 1-unit increase in NMLAR was associated with a 7% higher risk of CLRD-specific mortality and an 8% higher risk of all-cause mortality. RCS analysis demonstrated a linear dose–response relationship between NMLAR and mortality risk. The trend test based on quartile categorization further supported this finding, showing a progressive increase in HR from Q1 to Q4. The optimal thresholds derived from the Youden index were 3.91 for CLRD-specific mortality and 4.57 for all-cause mortality. Based on the above thresholds, risk stratification analysis revealed that high-risk individuals had substantially elevated risks of CLRD-specific mortality compared with their low-risk counterparts (overall: 2.12-fold; generalized COPD subgroup: 2.16-fold; asthma subgroup: 2.63-fold). In contrast, the magnitude of increase in all-cause mortality among high-risk individuals was relatively smaller (1.28-fold, 1.35-fold, and 1.47-fold, respectively), suggesting that NMLAR may have greater discriminatory value for CLRD-specific mortality risk stratification than for all-cause mortality. This may be because NMLAR aligns more closely with the underlying pathophysiological mechanisms of chronic lower respiratory diseases, where systemic inflammation and nutritional decline play pivotal roles. As a composite index integrating inflammatory, immune, and nutritional dimensions, NMLAR directly reflects these processes, thereby providing greater discriminatory power for stratifying disease-specific mortality risk (Mall et al., 2023; Zinellu et al., 2021). In contrast, all-cause mortality encompasses a wide spectrum of death causes, many of which are only weakly associated with inflammatory status, thus diminishing the predictive contribution of NMLAR.

In further subgroup analyses, we additionally stratified participants by ACO status, comprising those with ACO and those with COPD or asthma alone, to explore potential heterogeneity. Overall, the positive association between elevated NMLAR and mortality risk was consistent across most subgroups, reinforcing its robustness as a prognostic indicator. However, significant heterogeneity was observed by age: among participants <60 years, each 1-unit increase in NMLAR was associated with a 20% higher risk of all-cause mortality, whereas the corresponding increase in older adults was only 6%. This suggests that NMLAR carries greater prognostic relevance in younger individuals, whose lower baseline mortality risk may render them more susceptible to the adverse effects of systemic inflammation and nutritional decline, while in older adults, the higher comorbidity burden and baseline risk may attenuate its incremental predictive value (Liu et al., 2019). For CLRD-specific mortality, the predictive effect of NMLAR was most pronounced in the ACO subgroup, with a significant interaction detected. The heightened sensitivity of ACO patients to NMLAR may reflect their greater systemic inflammatory burden and complex immune-inflammatory responses, which aligns with prior evidence that ACO patients have higher rates of exacerbations and worse outcomes compared with those with COPD or asthma alone (Polverino et al., 2024; Hashimoto et al., 2023; Wakazono et al., 2025). Taken together, these findings indicate that NMLAR provides stable prognostic information across diverse populations, but its predictive value is particularly notable in younger individuals and in patients with ACO.

NMLAR demonstrates considerable clinical value as an independent prognostic factor. In Model 1, which included only NMLAR, the time-dependent C-index for predicting 1-year CLRD-specific mortality exceeded 0.80, indicating moderate short-term discriminatory capacity. However, when compared to multivariable models incorporating NMLAR (Model 2 and Model 3), its predictive accuracy declined markedly over longer follow-up durations. This suggests that while NMLAR alone is useful for short-term risk assessment, its long-term prognostic utility may be limited, particularly in predicting all-cause mortality. The performance drop may be attributed to fluctuations in NMLAR caused by transient factors such as acute infections or nutritional changes, undermining its stability over time. In contrast, Model 3—integrating top-ranked machine learning–selected features—demonstrated superior accuracy and generalizability for both intermediate- and long-term prediction. DCA and bootstrap calibration curves confirmed that Model 3 offered the highest net clinical benefit and better calibration for both CLRD-specific and all-cause mortality (Wang S. et al., 2025). It consistently maintained the highest C-index, exceeding 0.90 during mid-to long-term follow-up. Furthermore, external validation using the NHANES 2015–2018 cohort substantiated the model’s robustness and generalizability. These findings underscore the value of machine learning–based feature selection in building clinically effective prognostic tools. As a core component of the final model, NMLAR contributed uniquely to mortality prediction in CLRD populations, significantly enhancing both the model’s discriminative performance and clinical utility.

In addition to multi-timepoint validation within NHANES, we further evaluated the prognostic performance of NMLAR in two ICU cohorts with distinct clinical contexts. In the large and heterogeneous MIMIC-IV cohort, NMLAR remained independently associated with mortality after adjustment for both NHANES-derived covariates and ICU-specific factors, with RCS analysis suggesting an overall linear relationship and threshold analysis identifying a cutoff of 12.1, above which mortality increased markedly. The Zhejiang Provincial ICU cohort, although smaller and more homogeneous, provided real-world validation in a Chinese single-center setting; despite limited adjustment for smoking status, NMLAR retained strong prognostic value for 30-day mortality, with a similar threshold of 13.32. The consistency of these thresholds across diverse populations supports the stability of NMLAR-based risk stratification, and the moderate discriminative performance of NHANES-derived models in both ICU cohorts further reinforces the transportability of the framework and the clinical utility of NMLAR as a prognostic biomarker in critically ill CLRD patients.

Using nationally representative NHANES 1999–2018 data, this study benefited from a large sample size, broad variable coverage, and standardized measurements. We developed the NMLAR index, integrating systemic inflammation and nutritional status, and distinguished CLRD-specific mortality through the NDI to enable disease-focused risk stratification. Data-driven feature selection (Boruta, SVM-RFE, XGBoost) and comprehensive model assessment (C-index, calibration, DCA) strengthened methodological rigor. External validation across NHANES 2015–2018, the MIMIC-IV v3.1 ICU cohort, and the real-world Zhejiang Provincial ICU cohort further confirmed the robustness and generalizability of our findings, including in models that transported NHANES-derived coefficients without refitting and were additionally adjusted for critical-care–specific variables. However, the study’s observational design limits causal inference; some variables were self-reported and may introduce bias; and findings from U.S. NHANES data may not fully generalize to other populations. Larger prospective cohorts are needed to further validate the CLRD-specific mortality model.

## Conclusion

NMLAR, a composite indicator reflecting systemic inflammation and nutritional status, was an independent predictor of mortality among adults with CLRD and demonstrated an approximately linear dose–response relationship. Its prognostic value was consistently validated across multiple cohorts, supporting NMLAR as a robust and broadly applicable tool for individualized risk stratification in CLRD, including critically ill populations. Moreover, this study provides important evidence supporting the utility of relative blood-cell proportions in risk assessment for chronic diseases. Collectively, these findings provide a conceptual reference for applying relative immune–cell proportion metrics to inflammatory disease risk assessment.

## Data Availability

The original contributions presented in the study are included in the article/Supplementary Material, further inquiries can be directed to the corresponding authors.

## Supplementary material

SUPPLEMENTARY FIGURE S1 Immune cell fractions in the AECOPD cohort (GSE60399). PBMC samples from stable COPD and AECOPD patients were analyzed by CIBERSORT at hospital days 1, 3, and 10.

SUPPLEMENTARY FIGURE S2 Boruta algorithm–derived variable importance for CLRD-specific mortality. The x-axis represents the mean importance score (mean Z-score), and the y-axis lists the retained features, ranked in descending order of importance. All features shown were confirmed as significant predictors of CLRD-specific mortality.

SUPPLEMENTARY FIGURE S3 Top 20 important features for CLRD-specific mortality identified by the XGBoost algorithm. The x-axis shows the mean gain, representing each variable’s relative contribution to the model, while the y-axis lists the ranked features in descending order of importance.

SUPPLEMENTARY FIGURE S4 Boruta algorithm–derived variable importance for all-cause mortality. The x-axis represents the mean importance score (mean Z-score), and the y-axis lists the retained features, ranked in descending order of importance. All features shown were confirmed as significant predictors of all-cause mortality.

SUPPLEMENTARY FIGURE S5 Top 20 important features for all-cause mortality identified by the XGBoost algorithm. The x-axis shows the mean gain, representing each variable’s relative contribution to the model, while the y-axis lists the ranked features in descending order of importance.

SUPPLEMENTARY FIGURE S6 External validation of NHANES models in the MIMIC cohort (7-,14-,30-,365-day mortality). RCS analysis demonstrated an approximately linear association between NMLAR and mortality, accompanied by good calibration and greater clinical net benefit on decision-curve analysis.
