# Supplementary figures and images for: Prognostic value of the relative neutrophil–monocyte-to-lymphocyte–albumin ratio in chronic lower respiratory diseases: a multicenter retrospective analysis
Source: Front Physiol. 2025 Dec 18;16:1708302. doi: 10.3389/fphys.2025.1708302 (PMC12756091; doi:10.3389/fphys.2025.1708302)

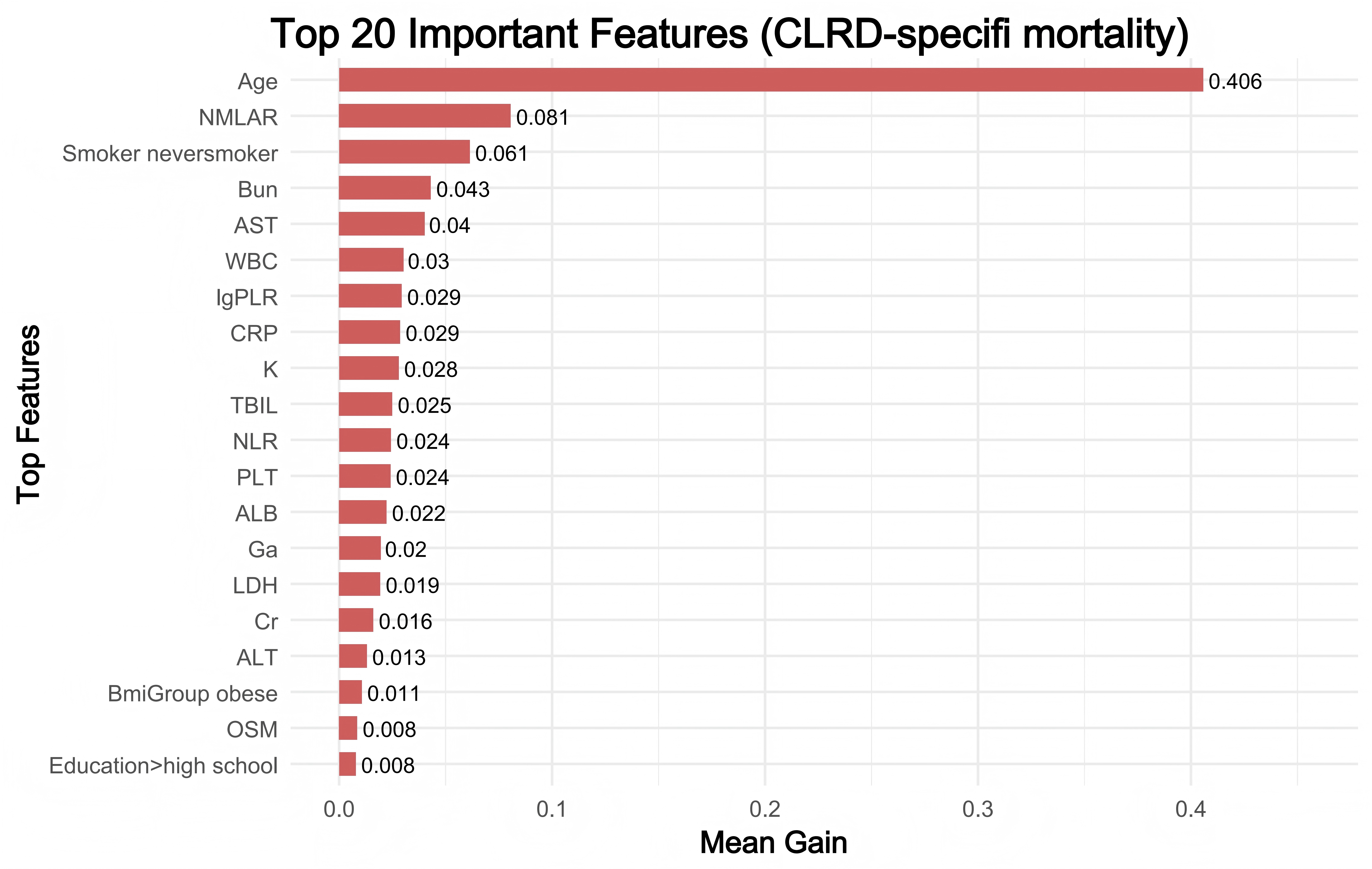

Supplement: Supplementary file 2 [file Image3.jpeg]

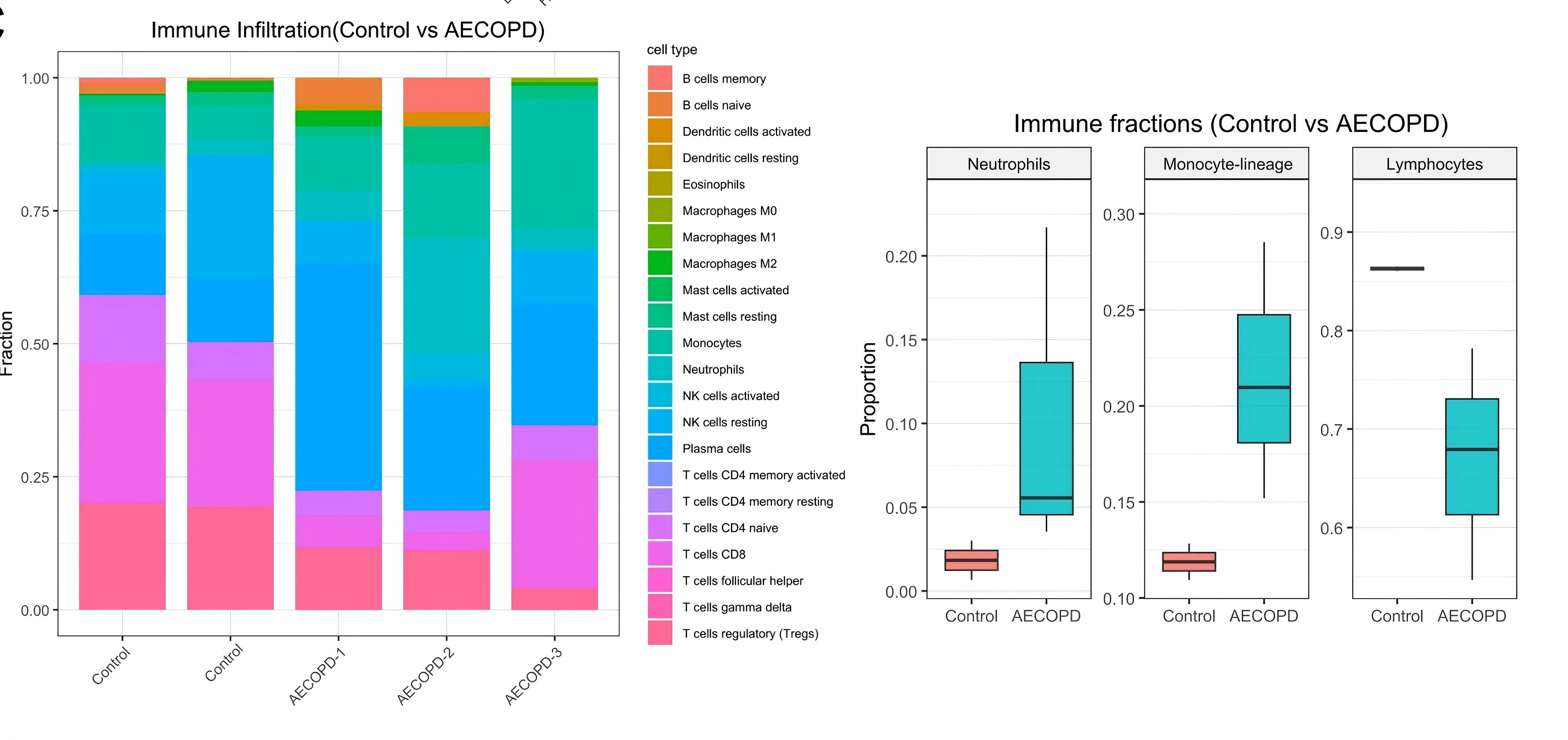

Supplement: Supplementary file 4 [file Image1.jpeg]

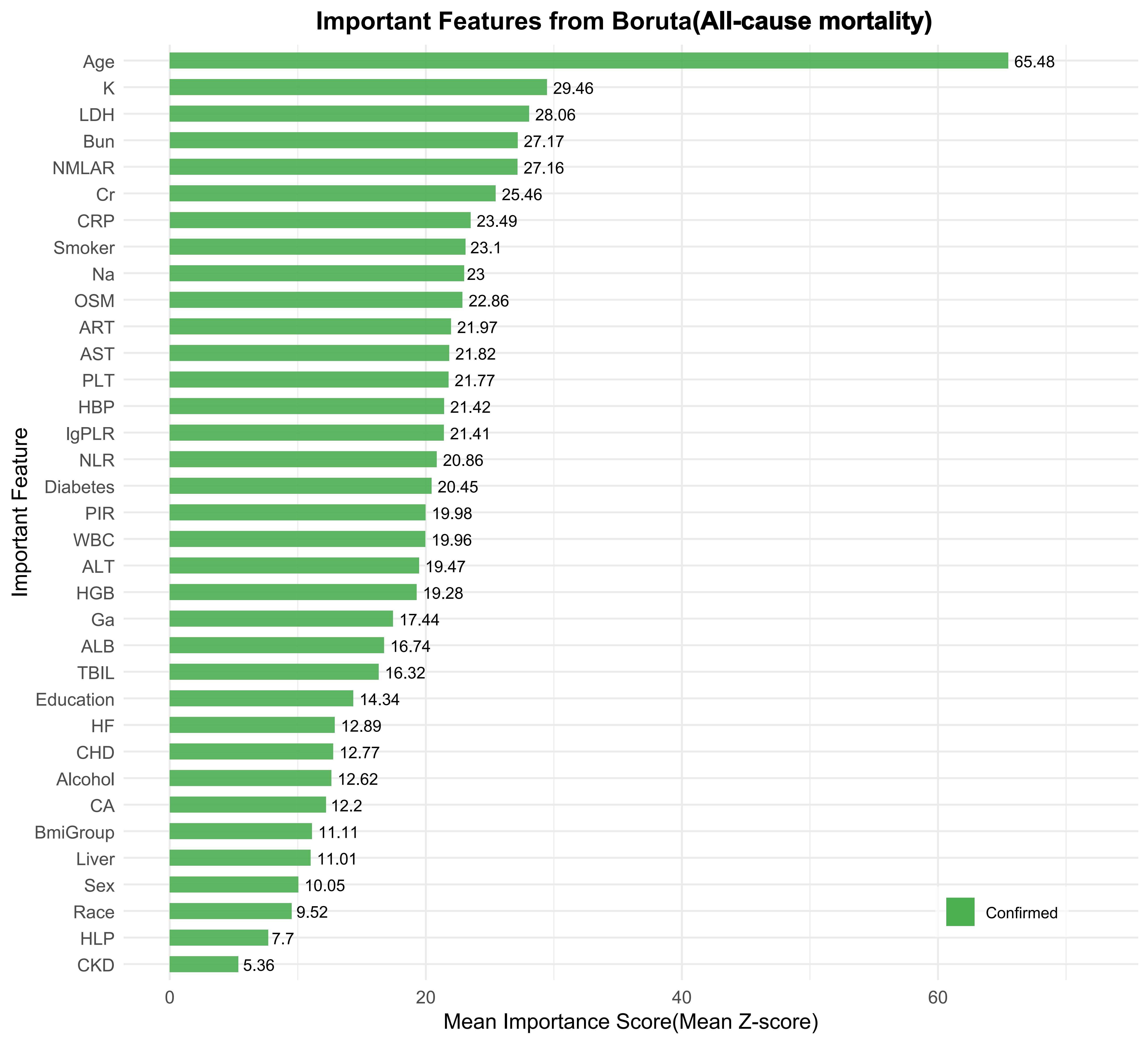

Supplement: Supplementary file 5 [file Image4.jpeg]

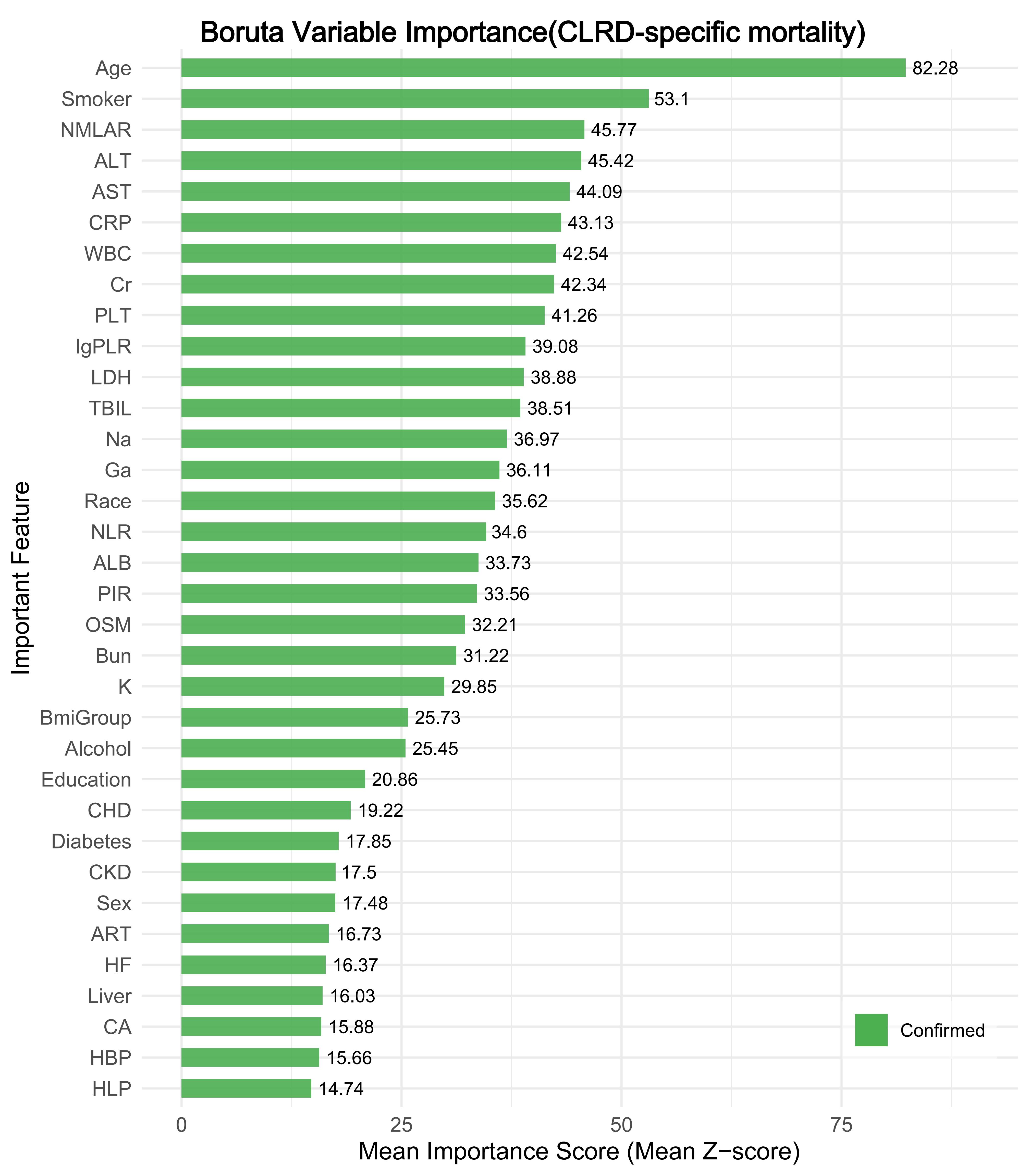

Supplement: Supplementary file 6 [file Image2.jpeg]

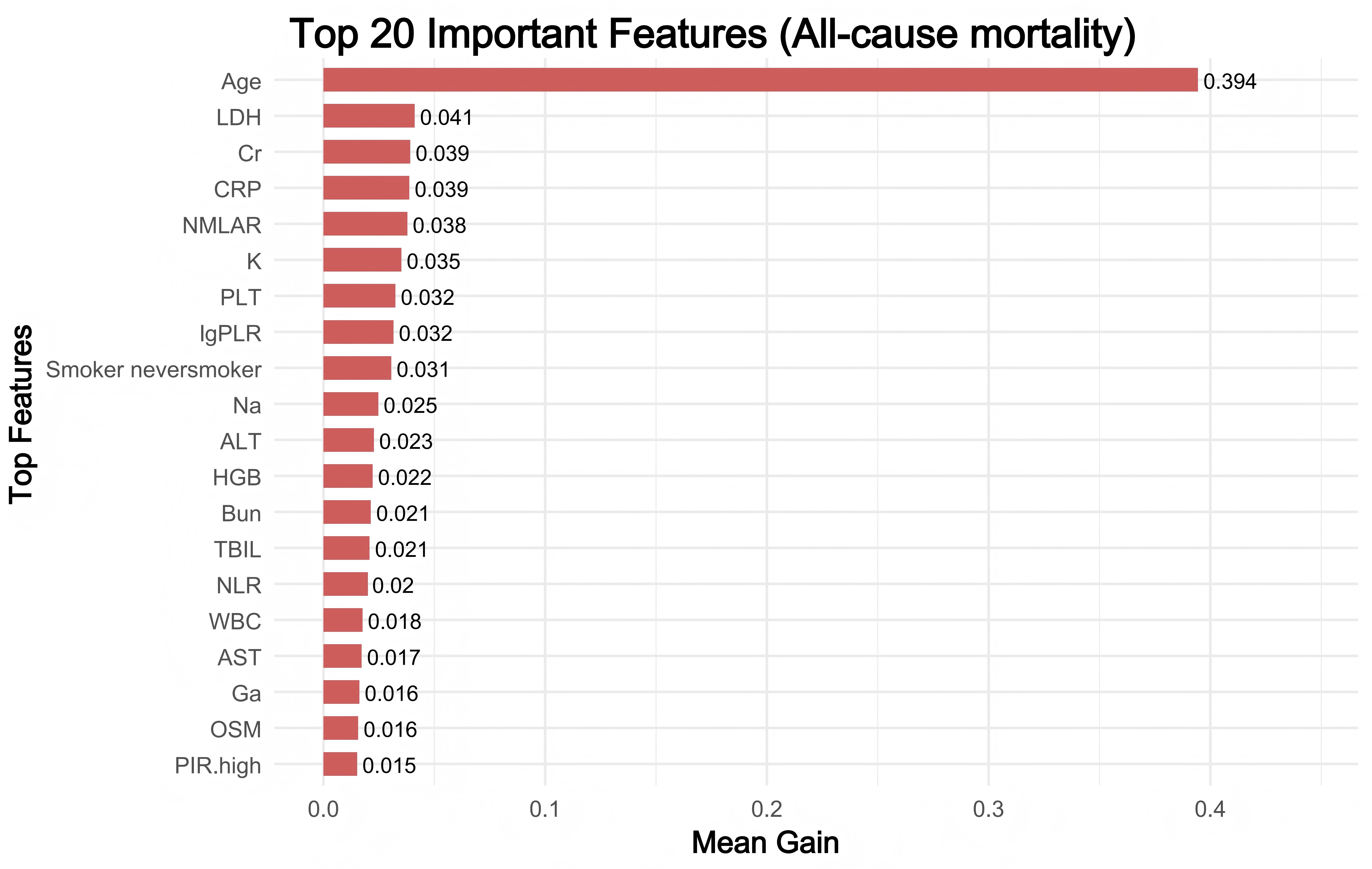

Supplement: Supplementary file 7 [file Image5.jpeg]

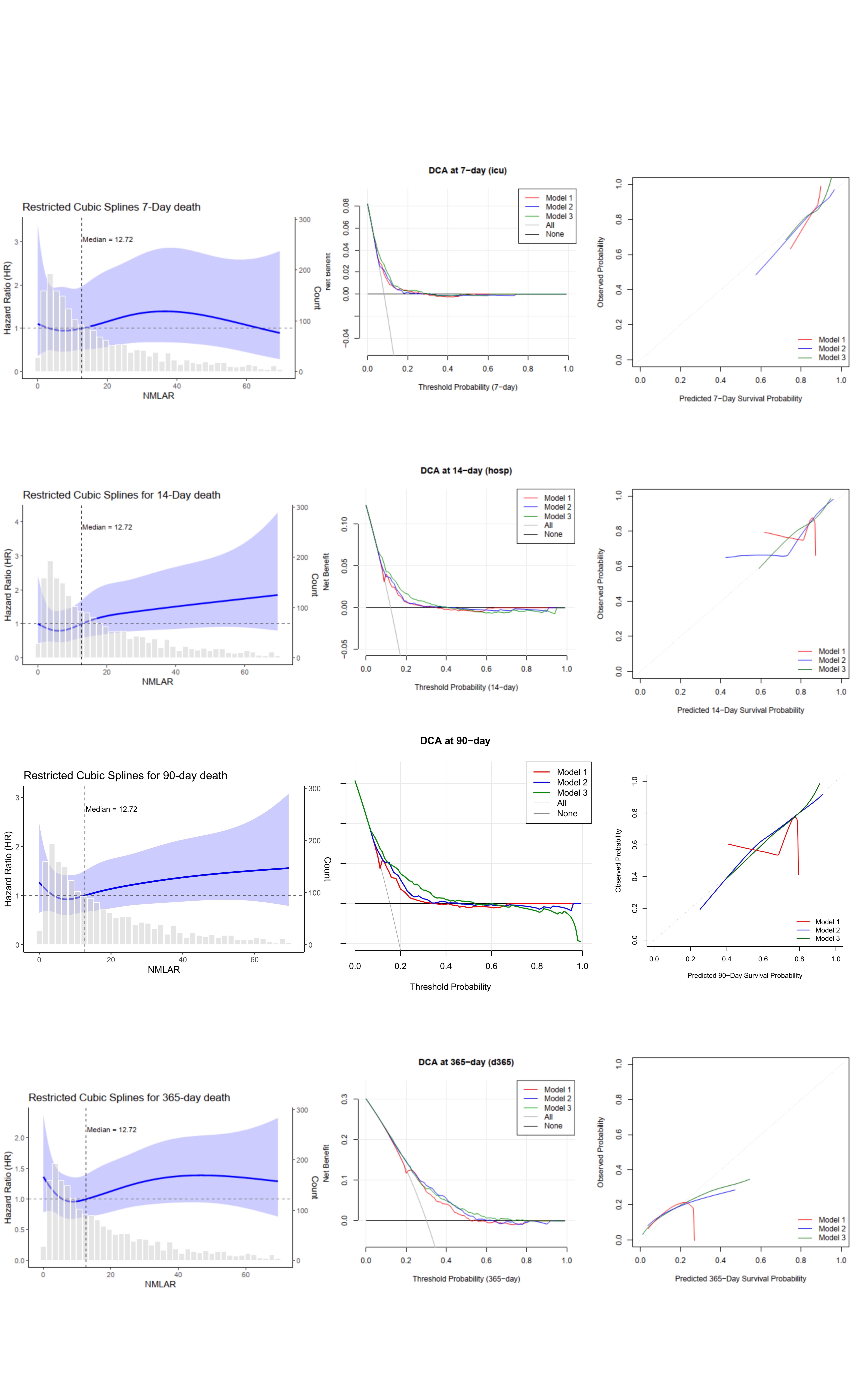

Supplement: Supplementary file 13 [file Image6.jpeg]
